# Correlated rotational switching in two-dimensional self-assembled molecular rotor arrays

**DOI:** 10.1038/ncomms16057

**Published:** 2017-07-04

**Authors:** Natalie A. Wasio, Diana P. Slough, Zachary C. Smith, Christopher J. Ivimey, Samuel W. Thomas III, Yu-Shan Lin, E. Charles H. Sykes

**Affiliations:** 1 Department of Chemistry, Tufts University, 62 Talbot Avenue, Medford, Massachusetts 02155, USA

## Abstract

Molecular devices are capable of performing a number of functions from mechanical motion to simple computation. Their utility is somewhat limited, however, by difficulties associated with coupling them with either each other or with interfaces such as electrodes. Self-assembly of coupled molecular devices provides an option for the construction of larger entities that can more easily integrate with existing technologies. Here we demonstrate that ordered organometallic arrays can be formed spontaneously by reaction of precursor molecular rotor molecules with a metal surface. Scanning tunnelling microscopy enables individual rotors in the arrays to be switched and the resultant switches in neighbouring rotors imaged. The structure and dimensions of the ordered molecular rotor arrays dictate the correlated switching properties of the internal submolecular rotor units. Our results indicate that self-assembly of two-dimensional rotor crystals produces systems with correlated dynamics that would not have been predicted *a priori*.

## Abstract (web summary)

Single molecular machines are capable of a variety of functions, but methods to couple motion between them are still lacking. Here, Wasio *et al*. report the emergent behaviour of spontaneously formed two-dimensional crystals, which display correlated switching of their sub-molecular rotor units.

Recent years have seen a proliferation of single-molecule devices including rotors^1^,^2^,^3^, motors^4^,^5^,^6^,^7^,^8^,^9^,^10^ and switches^11^,^12^,^13^,^14^,^15^,^16^,^17^,^18^,^19^,^20^,^21^. However, a key step towards realizing their full potential involves understanding and controlling the coupling between individual units. Basic computations and logic operations have been demonstrated in several surface-bound molecular systems, the earliest of which revealed that CO molecules could be arranged into specific configurations using a probe tip and one-time molecular cascades triggered to perform simple logic operations^22^. However, the assembly of this and more recent systems has relied on labour-intensive serial assembly of individual atoms or molecules with scanning probe tips^22^,^23^,^24^,^25^,^26^,^27^.

Herein we report the spontaneous self-assembly of two-dimensional molecular crystals that contain interacting submolecular rotor units and exhibit correlated rotational switching. The design of the molecules themselves enables ethyl groups, which behave as submolecular rotors, to have two preferred rotary positions pointing either towards or away from a surface, represented as a 0 or 1. We also show that self-assembled arrays with different packing structures exhibit different correlated switching behaviours.

## Results

### Self-assembly of two-dimensional molecular rotor crystals

**Figure 1:**
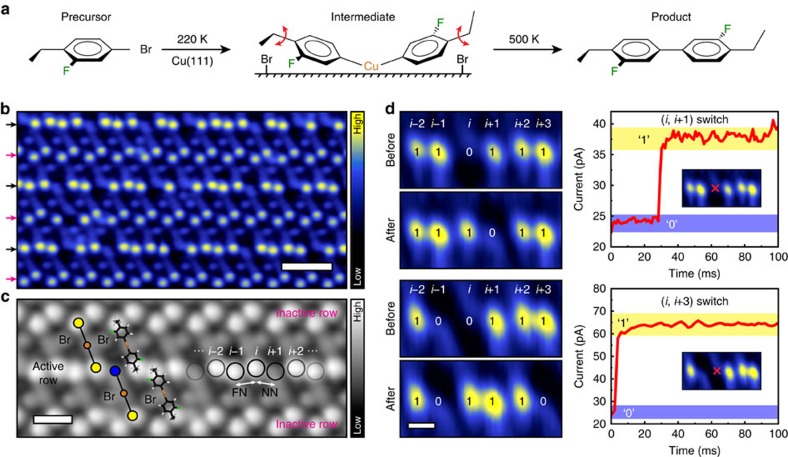
Correlated switching in self-assembled molecular rotor crystals. (**a**) Surface-mediated Ullmann coupling reaction scheme. (**b**) STM image of a two-dimensional crystal composed of the reaction intermediate in which the ethyl rotors appear either as bright protrusions or depressions depending on their orientation. The reaction intermediates form an interlocked pattern and the ethyl rotors form active (exhibiting correlated switching; black arrows) and inactive rows (no switching; magenta arrows). Scale bar, 2 nm. (**c**) Zoom in of the active row with both colour-coded and molecular overlays. Yellow and blue circles indicate the up and down configurations of the ethyl rotors, respectively. An example of ethyl rotor indexing is shown where *i*+1 and *i*−1 denote the NN and FN of rotor *i*, respectively. Scale bar, 1 nm. (**d**) Same-pair (*i*, *i*+1) and long-range (*i*, *i*+3)-correlated switching observed when pulsing rotor *i* in the active row with the STM tip. The resultant current versus time traces are shown with the location of the STM tip pulses (±50 mV) marked with a red cross; scale bar, 0.5 nm, and imaging performed at −10 mV and 5 pA.

In the case of the first molecule studied, 4-bromo-1-ethyl-2-fluorobenzene, at surface coverages of ∼0.3 monolayers, the Ullmann reaction intermediates self-assemble into two-dimensional crystals in which correlated interactions between the rotors lead to non-random switching sequences as shown in Supplementary Movie 1. A summary of the Ullmann coupling reaction scheme for the first molecular rotor of interest is shown in Fig. 1a. Briefly, upon annealing the Cu(111) surface, the C–Br bond in the precursor molecule is broken and an intermediate is formed that complexes with a surface Cu adatom^28^. These intermediates have ethyl tails at the extremity of the phenyl group that act as rotors and a geometry in which the phenyl groups are tilted away from the surface by ∼30° (ref. 29). Two-dimensional crystals of the intermediates spontaneously form during the reaction as shown in Fig. 1b–d, in which motion of the ethyl rotors is observed. The detached Br atoms are an integral part of the two-dimensional crystal and are labelled in Fig. 1c. If the surface is heated further, the reaction is completed by ejection of the Cu atom and formation of products^28^,^29^,^30^. Supplementary Figs 1–3 show further characterization of the Ullmann coupling reaction and self-assembly of the reaction intermediates of this majority crystal structure.

In Fig. 1b,c, it can be seen that the intermediates in these arrays form an interlocked pattern. The yellow and blue circles in Fig. 1c represent the up- and down-configurations of ethyl rotors, respectively. We only observe molecular motion of the ethyl rotors in every other row of the major crystal marked by the black arrows in Fig. 1b, whereas the magenta arrows highlight rows that do not exhibit motion. Control experiments (shown later in Fig. 3a) of reaction intermediates formed by reacting 1-bromo-3-fluorobenzene, the same molecule that lacks the ethyl rotor, confirm that the motion observed at the ends of the molecules is due to motion of the ethyl rotor. Within the active rows the ethyl rotors appear as either dark (ethyl down; 0) or bright (ethyl up; 1) features in the scanning tunnelling microscopy (STM) images with an apparent height difference of 46±3 pm. The ethyl rotors in both the active and inactive rows are in close contact with intermolecular distances alternating between near neighbour (NN: 0.46±0.02 nm) and far neighbour (FN: 0.60±0.03 nm) pairs in the active row. The inactive rows have a NN distance of 0.55±0.02 nm and a FN distance of 0.69±0.01 nm between ethyl rotors.

### STM tip-induced switching of individual rotors

Figure 1d shows experiments in which individual molecular rotors in the assembly were switched between 0 and 1 states and the effect on the neighbouring rotors. These experiments involved positioning the STM tip over a specific ethyl rotor to be excited (rotor *i* in Fig. 1d), pulsing at elevated voltage until a switch was observed in the current versus time (*I* versus *t*) trace, then imaging the surrounding array to reveal how the switching event affected neighbouring rotors. Two examples of tip-pulsing data (Fig. 1d) show that rotors labelled as *i*+1 and *i*+3 have switched concurrently with rotor *i*. Ethyl rotors in the active row are indexed such that *i*+1 refers to the NN of rotor *i* and *i*−1 refers to the FN. These tip-pulsing experiments were repeated, and the resulting types of correlated switches observed are shown in the histogram of Supplementary Fig. 4.

### Statistical analysis of correlated rotor switching in movies

In order to investigate these correlated switching events further, we perform time-lapse imaging of the arrays over periods of 14 h in which 1,724 switches were measured and analysed (see Supplementary Movies 1–2). By keeping the voltage and current low (less than ±50 mV, 10–50 pA) we could keep the excitation rate low so that the vast majority of the correlated motions would be detected. Experiments in which the STM tip was moved away from the two-dimensional rotor arrays for some time and then returned to the original area revealed that their motion is driven by the tip (see Supplementary Fig. 5 and Supplementary Note 1 for full details). Consistent with this, at 5 K the thermal energy available to the molecules is 0.43 meV, which is much lower than the calculated torsional barrier of 50 meV (Supplementary Fig. 6d). Furthermore, our previous work studying isolated molecular rotor intermediates formed from the reaction of a related molecule (1-bromo-4-ethylbenzene; molecular structure shown in Fig. 3c) with Cu(111) revealed that rotation of the ethyl groups could be excited by the STM tip above a threshold voltage ±45 mV, in agreement with the calculated torsional barrier. In the present study involving intermediates formed from 4-bromo-1-ethyl-2-fluorobenzene, we performed the same experiments and found that the ethyl rotor switching rate showed a strong dependence on the bias voltage and a weak dependence on tunnelling current (Supplementary Fig. 6a–c), consistent with electric field induced rotor switching via the STM tip^31^,^32^. The results of these experiments and a full discussion can be found in Supplementary Fig. 6 and Supplementary Note 2.

**Figure 2:**
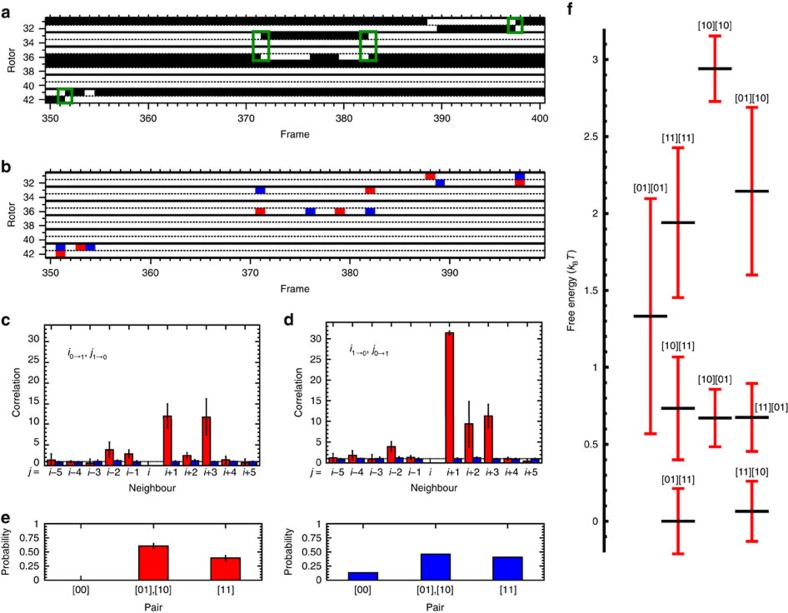
Statistical analysis of correlated switching. (**a**) Example on/off trajectory of the major crystal at −10 mV and 10 pA. White and black denote ethyl rotors pointing up (on; 1) and down (off; 0), respectively. Correlated switches are highlighted by green boxes. (**b**) Corresponding switching trajectory. Blue and red denote switching off (1→0) and switching on (0→1), respectively. Statistical analysis of 1,724 events reveals non-random switching and the predominance of several correlated switching events. The correlations plotted here are (**c**), the ratio of the conditional probability of an ethyl rotor at position *j* (*j*=*i*−5, …, *i*−1, *i*+1, …, *i*+5) switching off (1→0), given that the ethyl rotor at position *i* switches on (0→1), to the average probability of an ethyl rotor switching off (1→0) and (**d**) the ratio of the conditional probability of an ethyl rotor at position *j* switching on (0→1), given that the ethyl rotor at position *i* switches off (1→0), to the average probability of an ethyl rotor switching on (0→1). The relative position of *j* to *i* is defined so that *i*+1 is the NN of rotor *i*, while *i*−1 is the FN of rotor *i*. The red and blue bars are results from experiment and from simulation of random switches, respectively. (**e**) Histogram of experimentally observed populations of [00], [01]/[10] and [11] (left, red), compared to a binomial distribution calculated with an optimized off probability of 0.36275 (right, blue). (**f**) Free energies of the nine most populated [*ab*][*cd*] microstates. Error bars are the weighted s.d. Similar free energy diagram using three-pair microstates [*ab*][*cd*][*ef*] can be found in Supplementary Fig. 20.

Figure 2 shows a summary of statistical analysis of rotor switching events in the major crystal and full details of this analysis are given in the Supplementary (all rows of the major crystal are shown in Supplementary Fig. 7). Figure 2a,b shows how data from the STM movies are converted into on/off trajectories and the associated switching behaviours (all frames of the trajectory are shown in Supplementary Fig. 8). These data revealed that the ethyl rotors along the active rows exhibit both direct, correlated [10]⇔[01] switching within NN pairs (between rotors *i* and *i*+1) as well as long-range [1*x*][*y*0]⇔[0*x*][*y*1]-correlated switching (between rotors *i* and *i*+3). Here [*ab*] denotes a NN pair, and *a* and *b* can be 0 or 1. These correlated switches occur more often than expected if the switching was random as shown in Fig. 2c,d and are not dependent on scanning direction (additional correlations are shown in Supplementary Fig. 9). In contrast, the motion of the ethyl groups in one active row was not correlated with the other active rows, meaning that the two-dimensional crystals are essentially assemblies of highly correlated one-dimensional rotor chains that do not interact with each other.

We found that the average population of ethyl rotor up (1) versus down (0; *p*_1_ versus *p*_0_) was 70:30±2%, with an average switching probability per frame of 0.0192±0.0028. Comparing the populations for each [*ab*] configuration to the binomial distribution, calculated simply using *p*_0_ and *p*_1_, shows that the [00] configuration is highly unfavourable, compared to the distribution with the optimized *p*_0_ (Fig. 2e). We also analysed ∼25,000 switches of ∼5,000 molecular rotors in a number of crystals with identical packing structure. The observations that ethyl rotors highly disfavour the [00] configuration and show correlated (*i*, *i*+1) and (*i*, *i*+3) switches are emergent properties of this major type of crystal packing.

We further analysed the up–down configurations by grouping two pairs of NN ethyl groups [*ab*][*cd*], resulting in 2^4^=16 possible up–down configurations (hereafter termed microstates). Two-pair microstates were chosen here because they are the minimal number required to explain the observed (*i*, *i*+3) correlated switching. Comparing to a random array of 1 and 0 states with a 70:30 population, we found that certain microstates appeared more often and that these microstates remained dominant after hours of excitation by the STM tip. Figure 2f shows how these microstates order in terms of their relative free energies, which were calculated using their populations observed in experiment and the Boltzmann equation (see in Supplementary Fig. 10 for additional details). We find that the relative free energy ordering of these microstates does not change during an experiment, meaning that the system is at steady state. The commonly observed (*i*, *i*+1) and (*i*, *i*+3)-correlated switches resulted from exchanges among the microstates with low free energies. For example, [01][11]⇔[11][10] led to simultaneous switching of *i* and *i*+3, while [01][11]⇔[10][11] led to simultaneous switching of *i* and *i*+1, where underlines indicate switching rotors. Full details of the switching analysis can be found in Supplementary Note 3.

### Monte Carlo simulations

We also performed Monte Carlo simulations using these microstate energy levels to estimate the change of energy before and after a switch (see Supplementary Note 4 for more details). These Monte Carlo results qualitatively recapitulate both the two-pair microstate populations (Supplementary Fig. 11a) and the observed (*i*, *i*+1) and (*i*, *i*+3)-correlated switching behaviours (Supplementary Fig. 9a–d versus Supplementary Fig. 11b–e), showing that the transitions between microstates are principally based on their free energy diagram. This suggests that different ordering of the microstates generated by different self-assemblies can serve as a way to promote correlated switches with distinct patterns. For instance, we almost never observe the [00] configuration in this two-dimensional crystal, suggesting that such a configuration is highly unfavourable. Therefore, when ethyl rotor *i* in a [10] configuration switches from 1→0, its nearest neighbour must switch from 0→1 (Fig. 2d). However, when ethyl rotor *i* in a [01] configuration switches from 0→1, its nearest neighbour can remain at 1 or switch from 1→0 (Fig. 2c). In addition, since [00] is forbidden, the (*i*, *i*+3) correlated switching [1*x*][*y*0]⇔[0*x*][*y*1] only happens when the two middle rotors are up (1), that is, both *x* and *y* must be 1. Therefore, because [00] is forbidden, a [11] configuration is required to enable (*i*, *i*+3) correlated switches, [11][10]⇔[01][11].

### Analysis of correlated switching in other rotor crystals

**Figure 3:**
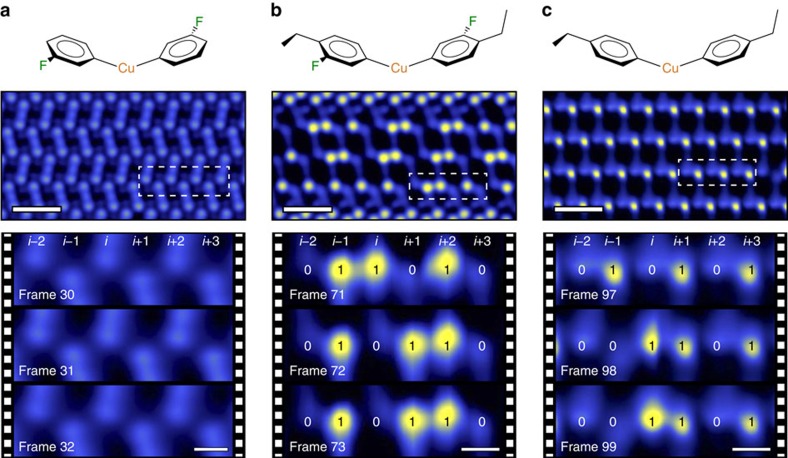
Effects of molecular structure on crystal packing and rotational switching. (**a**) The intermediate formed by reacting 1-bromo-3-fluorobenzene with the Cu(111) surface lacks an ethyl rotor and exhibits no switching events. (**b**) Minority rotor crystal structure with a different packing arrangement displays only short-range correlated (*i*, *i*+1) switching. (**c**) Removal of the fluorine atom (1-bromo-4-ethylbenzene precursor) produces another crystal structure in which (*i*, *i*−1)-correlated switching is observed. Scale bars for the top and bottom three panels, 2 nm and 0.5 nm, respectively.

Our observations of correlated switching in the active rows and no motion in the inactive rows, which have different ethyl–ethyl spacing, indicate that the spatial arrangement of the rotors is responsible for the correlated switching behaviours^13^,^21^. In order to test this hypothesis, we performed experiments on a minority crystal structure of the same surface intermediate as shown in Fig. 1; the results are shown in Fig. 3b (all rows are shown in Supplementary Fig. 12). This arrangement, while similar to the majority crystal structure, has different ethyl–ethyl spacings of 0.54±0.02 nm (NN) and 0.62±0.03 nm (FN). We applied the same analysis to molecular rotor switching in this crystal and found that there was a different energy landscape for the microstates, and that only (*i*, *i*+1) correlated switching occurred (see Supplementary Figs 13 and 14). We found that the average ethyl rotor up (1) versus down (0) was 50:50±1% in this crystal, with one up and one down in each NN pair (Supplementary Fig. 15). This suggests that both [00] and [11] configurations are highly unfavourable in the minority crystal, as compared to only [00] being highly unfavourable in the major crystal. Since [11] is required for (*i*, *i*+3)-correlated switches of [11][10]⇔[01][11], the prohibition of [11] in this crystal packing thus prevents the (*i*, *i*+3)-correlated switches from occurring. Details for the switching analysis of the minority crystal structure can be found in Supplementary Note 5.

## Discussion

While computational two-dimensional crystal engineering is not yet at the point that one can *a priori* predict relationships between molecular structure and crystal packing, we demonstrate experimentally in Fig. 3c that subtly changing the nature of the precursor molecule (F atom replaced by H) changes the packing structure. Reaction intermediates formed from 1-bromo-4-ethylbenzene form a different two-dimensional crystal structure (NN of 0.49±0.02 nm and FN of 0.54±0.02 nm, Supplementary Fig. 16) with different switching properties, resulting in only (*i*, *i*−1)-correlated switches (Supplementary Figs 17–19 and Supplementary Note 6). This result supports our hypothesis that the spatial arrangement of the rotors dictates their interactions and hence their collective function. The switching behaviours of all the crystal structures studied can be viewed in Supplementary Movie 2.

Major challenges in the continued miniaturization of devices include the size scale of top down lithography, signal degradation via electron tunnelling and power usage in terms of both battery limitation and heat dissipation^33^. We show here that nanoscale crystalline molecular rotor arrays can be formed spontaneously and exhibit correlated switching behaviour via the motion of coupled submolecular components rather than the movement of electrons. These are highly emergent properties because individual, isolated molecular rotor units show random rotational behaviour, whereas when coupled together in a self-assembled two-dimensional crystal, correlated rotational switching is observed that would not have been predicted from the behaviour of the individual units. The systems reported here are operated at low temperature; however, the barriers could be tuned via the design of the molecules themselves^3^. While a lot is known about structure–function relationships in three-dimensional crystals, two-dimensional crystal engineering is still in its infancy. Moving forward, by measuring the properties of two-dimensional crystals and then making subtle changes with organic synthesis, structure–function relationships can be drawn and emergent properties, like in this case correlated rotor switching, understood. While a variety of single-molecule devices have been reported, this approach offers a new direction for the field of molecular machines, providing ways to couple molecular motion between units and perform higher tasks.

## Methods

### STM experiments

STM experiments were performed in an Omicron Nanotechnology GmbH low-temperature scanning tunnelling microscope with a base pressure of <1 × 10^−11^ mbar. A Cu(111) single crystal (MaTecK GmbH) was cleaned by cycles of Ar^+^ bombardment and annealing to 1,000 K. Cleanliness of the crystal was determined by STM prior to molecular deposition. The precursor molecule 4-bromo-1-ethyl-2-fluorobenzene (>95%) was purchased from Matrix Scientific and degassed by several freeze–pump–thaw cycles. Additional precursor molecules, 1-bromo-3-fluorobenzene (>99%) and 1-bromo-4-ethylbenzene (97%), were purchased from Sigma-Aldrich and also underwent several freeze–pump–thaw cycles prior to use. Each molecular species was introduced into the ultrahigh vacuum chamber via a precision leak valve and vapour deposited onto the Cu(111) crystal held at 5 K in the STM stage. After deposition, the cooled crystal was removed from the stage and annealed to 220 K to initiate C–Br cleavage and to form highly ordered two-dimensional crystalline arrangements of the metal–organic intermediates. The sample was cooled back to 5 K for STM imaging and *I* versus *t* measurements. STM images were acquired at constant current with chemically etched W tips with the bias voltage applied to the sample. Imaging conditions for figures in the main text are as follows: −10 mV and 10 pA (Fig. 1b), +10 mV and 100 pA (Fig. 1c), −10 mV and 5 pA (all images in Fig. 1d), +10 mV and 100 pA (Fig. 3a top panel), +30 mV and 100 pA (Fig. 3a lower panel), −10 mV and 30 pA (all images in Fig. 3b), −30 mV and 30 pA (all images in Fig. 3c).

### Statistical analysis

Five hundred consecutive frames containing four active rows were used to analyse the switching behaviours of the major crystal packing formed by reacting 4-bromo-1-ethyl-2-fluorobenzene with Cu(111). The time series of each of the four rows and the associated switching series were used to calculate the populations of down (0) and up (1) (*p*_0_ and *p*_1_), the switching probabilities of 0→1 and 1→0 (*s*_0_→_1_ and *s*_1_→_0_) and the overall switching probability. For comparison, a random data set containing the same number of rows and rotors with matching *p*_0_, *p*_1_ and *s*_0_→_1_ was also generated and analysed. The correlation was then calculated to quantify the correlation patterns between the observed simultaneous switches. When simultaneous switching occurred for ethyl rotors *i* and *j* from frame *N* to *N*+1, their correlation pattern was categorized as (A) *i* switched from 0→1 but *j* switched from 1→0 and (B) *i* switched from 1→0 but *j* switched from 0→1. The relative position of ethyl rotor *j* to *i* (or *i* to *j*) was further defined by choosing the direction towards the NN of *i* (or *j*) as the positive direction. The conditional switching probabilities of *i*+*n*, given *i* switched, were then normalized by the average *s*_0_→_1_ (when *i*+*n* switched on) or *s*_1_→_0_ (when *i*+*n* switched off) calculated using all four rows to compute the correlations. Using the correlations from the four rows, weighted average and s.d. (weighted by the number of ethyl rotors in each row) were calculated, and compared with those calculated from the random data set. To determine the populations of the one-pair states, each row was broken down into sets of one NN pair. Using the populations of one-pair states from the four rows, weighted average and s.d. (weighted by the number of one-pair states in each row) were calculated. Subsequently, the off (0) probability was optimized to give a binomial distribution that minimizes the sum of the difference squares between the experimental and theoretical distributions. To determine the two-pair ground state, each row was first broken down into sets of two NN pairs [*ab*][*cd*] (a total of four ethyl rotors), analysed with a sliding window. For sets of two NN pairs [*ab*][*cd*], there were a total of 2^4^=16 microstates. The population of each of the 16 microstates was calculated for each row. To calculate the total average population for each of the 16 states, the populations obtained from the four rows were averaged and weighted by the number of two-pair sets each row had. Subsequently, the relative free energy of each state was calculated, using the highest populated two-pair state ([01][11]) as the reference.

### Data availability

The data that support the findings of this study are available from the corresponding author upon request.

## Additional information

**How to cite this article:** Wasio, N. A. *et al*. Correlated rotational switching in two-dimensional self-assembled molecular rotor arrays. *Nat. Commun.* **8,** 16057 doi: 10.1038/ncomms16057 (2017).

**Publisher’s note:** Springer Nature remains neutral with regard to jurisdictional claims in published maps and institutional affiliations.

## Supplementary Material

Supplementary Movie 1 Supplementary Figures and Notes.

Supplementary Movie 2 80 frames of a 575 frame movie showing the correlated rotational switching events occurring in the active rows of the major crystal structure formed from the reaction of 4-bromo- 1-ethyl-2-fluorobenzene with Cu(111). Frames were acquired at -10 mV and 30 pA and rendered at a frame rate of 2 frames per second.

Supplementary Information Rotational switching events in different molecular crystal packing structures. Frames were acquired at -10 mV and 30 pA (major), -10 mV and 10 pA (minor), -30 mV and 30 pA, and +30 mV and 100 pA for surface reaction intermediates of 4-bromo-1-ethyl-2- fluorobenzene, 1-bromo-4-ethylbenzene, and 1-bromo-3-ethylbenzene, respectively. The frame rate of the minor crystal structure was increased to 8 frames per second due to a lower switching probability; all other movies are rendered at 2 frames per second.
