# Supplementary material for: Correlated rotational switching in two-dimensional self-assembled molecular rotor arrays
Source: Nat Commun. 2017 Jul 4;8:16057. doi: 10.1038/ncomms16057 (PMC5500884; doi:10.1038/ncomms16057)
Supplement: Supplementary Information — Rotational switching events in different molecular crystal packing structures. Frames were acquired at -10 mV and 30 pA (major), -10 mV and 10 pA (minor), -30 mV and 30 pA, and +30 mV and 100 pA for surface reaction intermediates of 4-bromo-1-ethyl-2- fluorobenzene, 1-bromo-4-ethylbenzene, and 1-bromo-3-ethylbenzene, respectively. The frame rate of the minor crystal structure was increased to 8 frames per second due to a lower switching probability; all other movies are rendered at 2 frames per second. [file ncomms16057-s3.pdf]

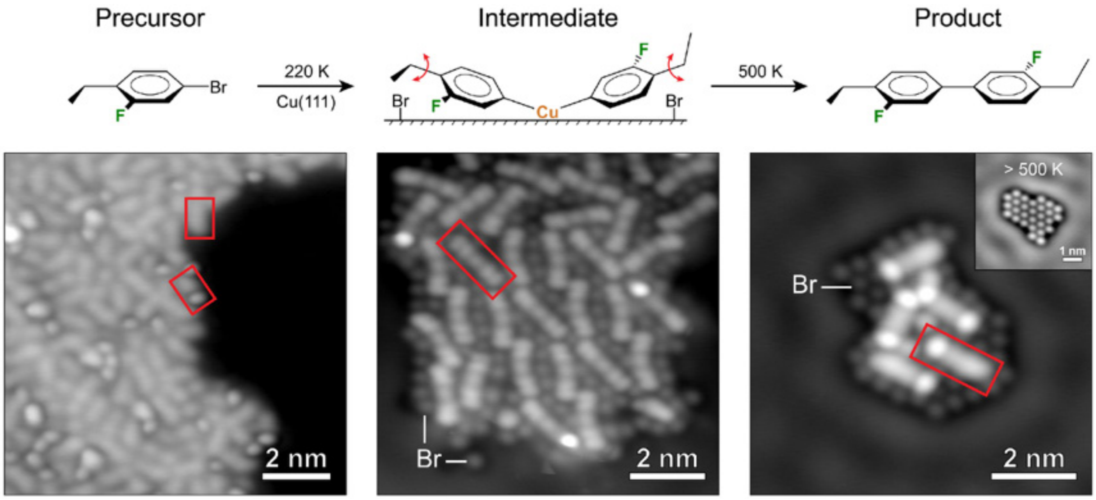

Supplementary Fig. 1. Progress of the surface mediated Ullmann coupling reaction using STM at 5 K. Precursor molecules (4-bromo-1-ethyl-2-fluorobenzene) are dosed on a Cu(111) surface and annealed to 80 K, shown in the left panel. Image contrast can differ within individual molecules (highlighted with two red boxes) depending on the orientation of the ethyl moiety. Annealing to 220 K produces the surface-bound reaction intermediate (red box) and surrounding dissociated Br atoms; middle panel. Bright features indicate the ethyl rotors pointing toward the STM tip. Product formation occurs when annealing to 500 K (red box); right panel. Inset: Only Br atoms remain after product desorption during further annealing. Scan conditions for precursor, intermediate, and product panels, respectively: +10 mV and 50 pA, +15 mV and 20 pA, +10 mV and 100 pA, and +10 mV and 300 pA (inset).

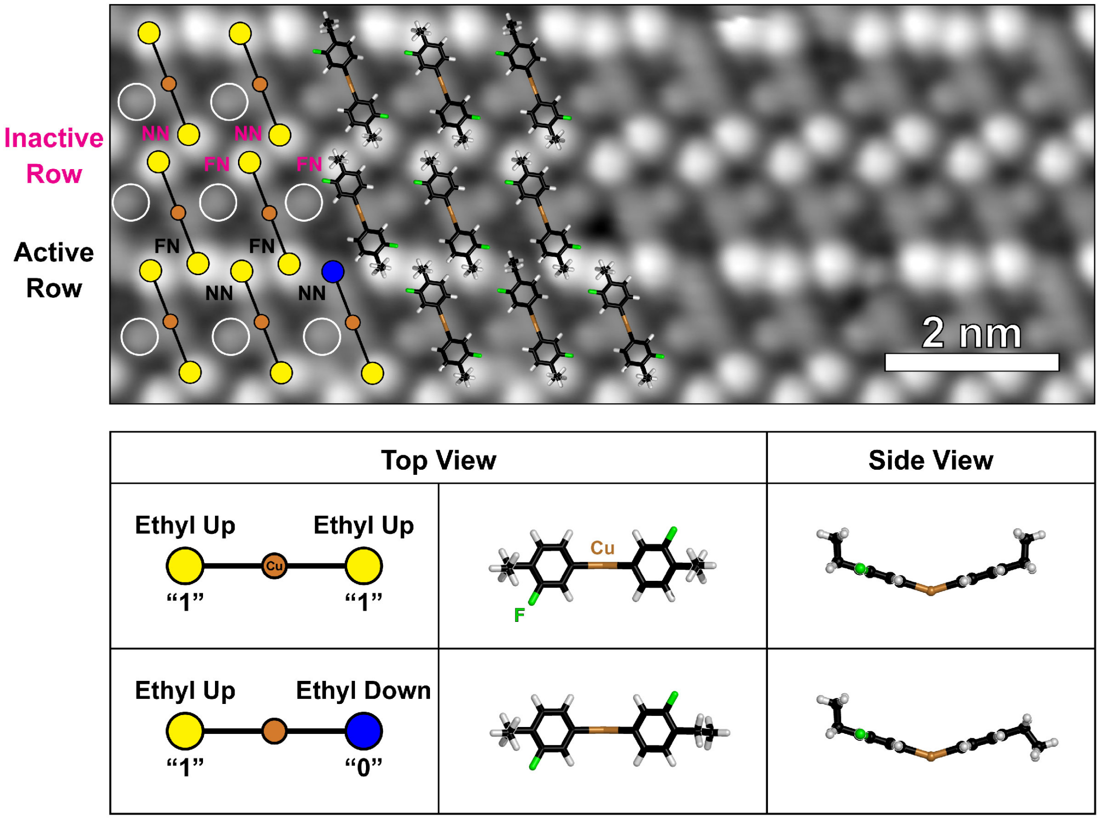

	Active Row	Inactive Row
**NN**	0.46 ± 0.02 nm	0.55 ± 0.02 nm
**FN**	0.60 ± 0.03 nm	0.69 ± 0.01 nm

Supplementary Fig. 2. Interlocking pattern of the reaction intermediates in the major crystal with generic and molecular overlays. Dissociated Br atoms are part of the crystal array and are highlighted with white circles. The generic overlays are color-coded to indicate the ethyl orientation and are summarized in the middle panel. Both active and inactive rows alternate between near neighbor (NN) and far neighbor (FN) distances as indicated in the top panel and average distances summarized in the bottom panel. Scan condition for top panel: +10 mV and 100 pA.

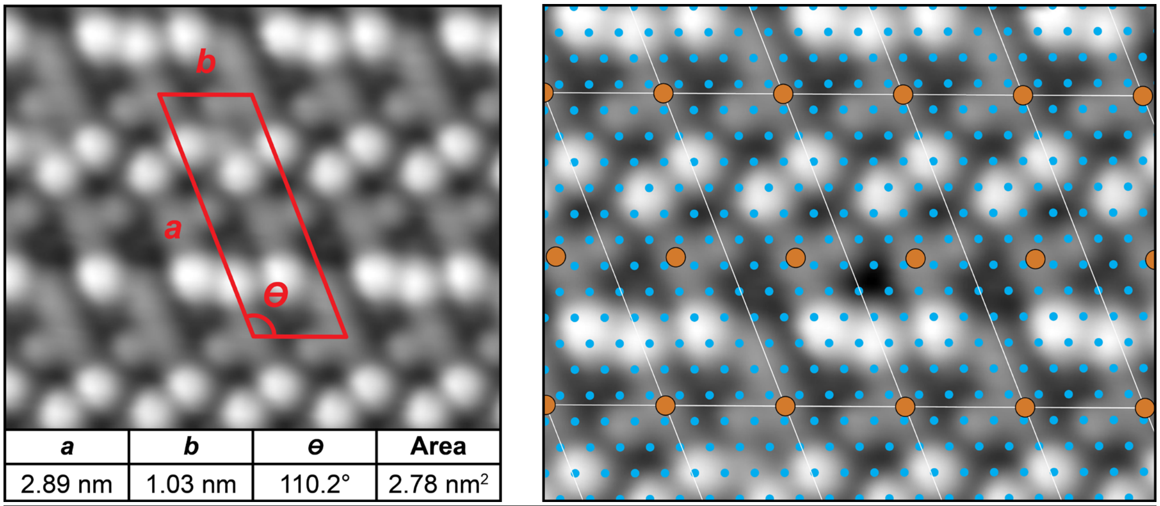

a	b	θ	Area
2.89 nm	1.03 nm	110.2°	2.78 nm^2

Supplementary Fig. 3. Left: Unit cell dimensions of the major crystal structure. Right: Same unit cell structure as shown on the left with brown circles marking the copper atom centers of the reaction intermediates and blue circles as the Cu(111) lattice. A coincident Cu(111) lattice with size of 2.84 nm × 1.02 nm agrees well with experimental values (± 0.05 nm) as determined by 2D FFT and places the Cu atom at the center of each organometallic complex at a three-fold Cu site. Scan condition: +10 mV and 100 pA.

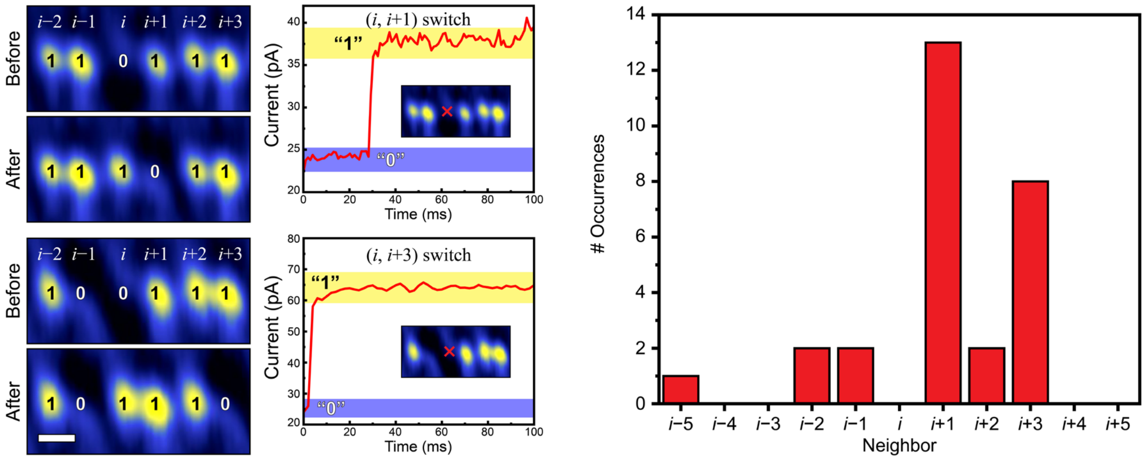

Supplementary Fig. 4. Left: Pulsing the major crystal structure as shown in Fig. 1d in the main text. The STM tip was positioned over rotor *i* (also shown as a red cross) and a ±50 mV pulse was applied for 100 ms. The current during the tip pulse was monitored over time in which rotor *i* switched from 0 to 1. During these experiments both (*i*, *i*+1) and (*i*, *i*+3) switches are observed and are the predominant events when the crystal is pulsed with the STM tip numerous times, as shown in the histogram on the right.

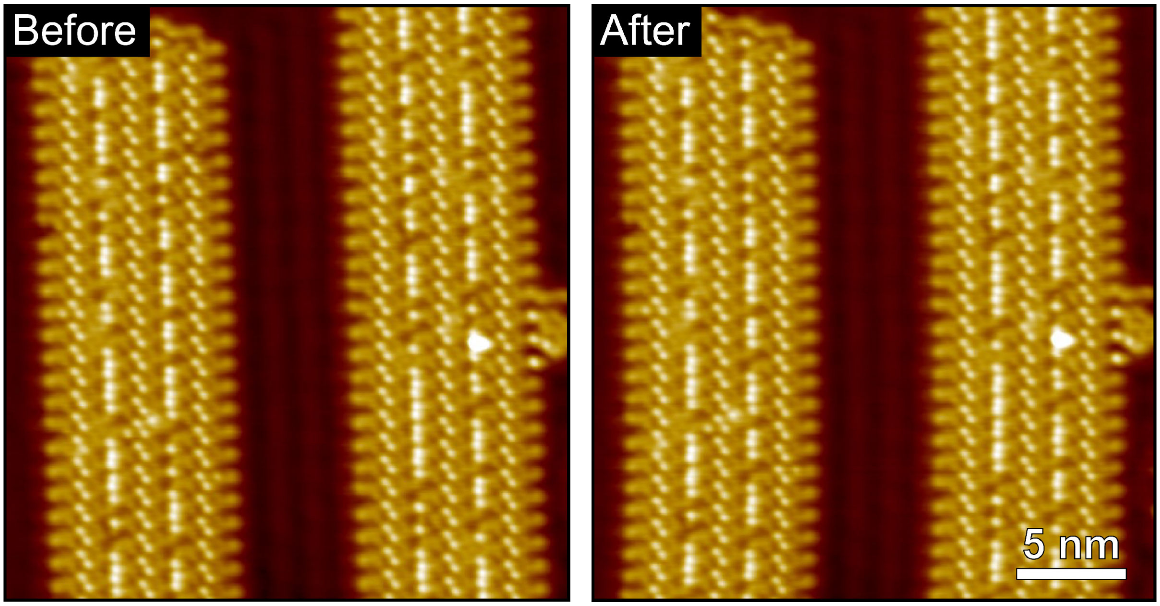

Supplementary Fig. 5. Control experiments to rule out thermal effects in switching in the major crystal array. The rotor array was imaged before and after the STM tip was moved 100 nm away for 10 minutes.

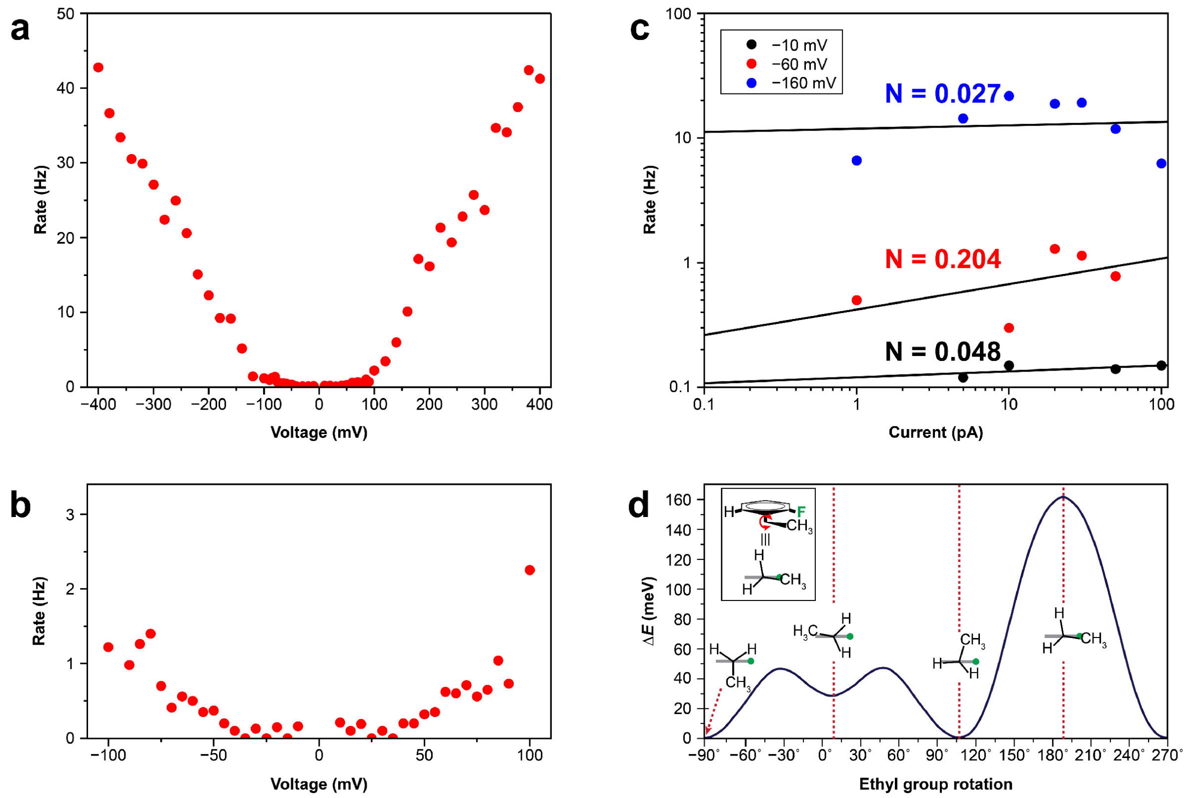

Supplementary Fig. 6. a–c, Switching rate as a function of bias voltage and current. The lines in panel **c** are a power law fit to the data (rate ∝ I^N). **d**, Calculated torsional potential for ethyl group rotation with two energy minima corresponding to ethyl rotor up and down orientations. The energy of the gas-phase 4-bromo-1-ethyl-2-fluorobenzene was calculated at 1° increments of rotation of the ethyl group using the Gaussian 09 software package with the B3LYP functional and the 6-311+G(d,p) basis set[1].

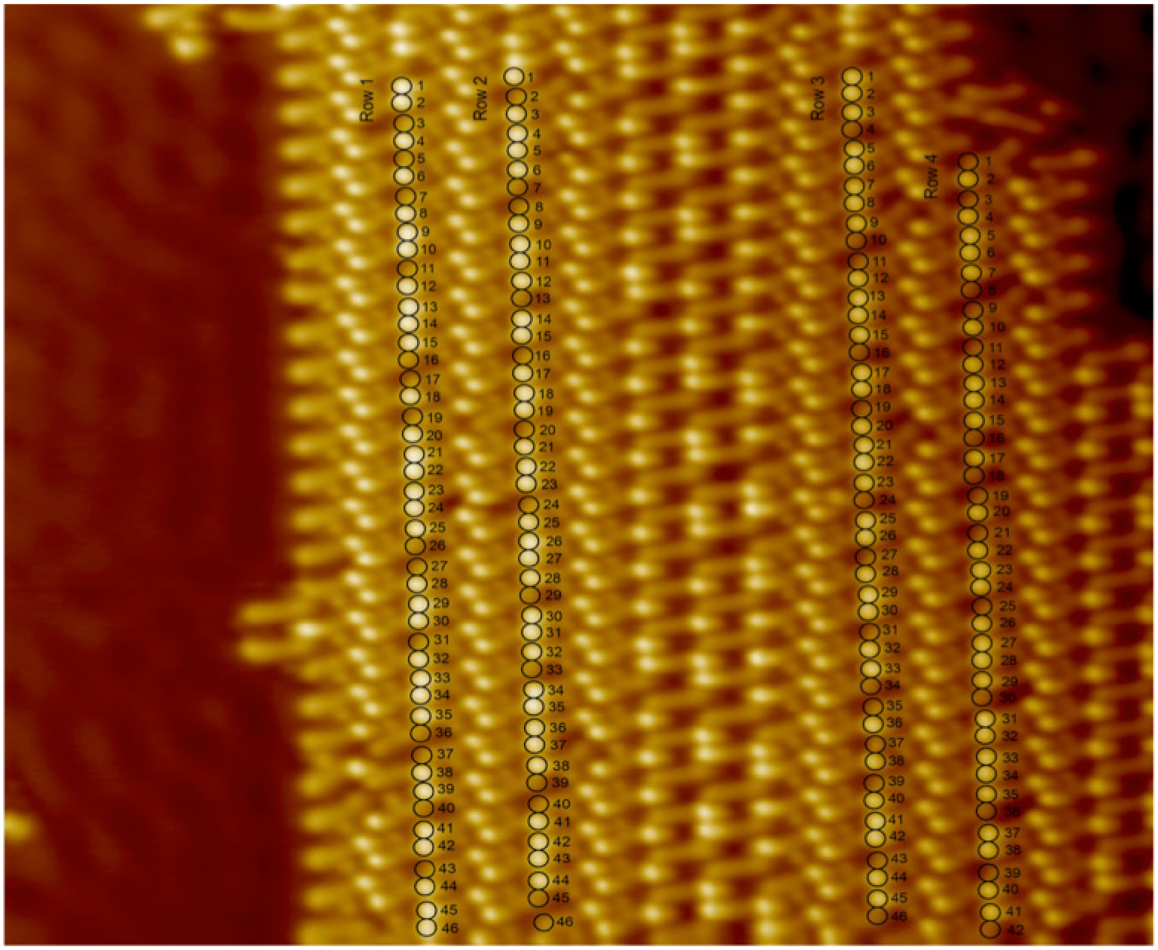

Supplementary Fig. 7. STM image of the major crystal packing formed by reacting 4-bromo-1-ethyl-2-fluorobenzene with Cu(111). The ethyl rotors appear either as bright protrusions or depressions depending on their orientation. The ethyl rotors are numbered for each of the four rows analyzed. The image size and scanning conditions are 28 nm × 28 nm and −10 mV and 10 pA, respectively.

a. Row 1 $p_0 = 32.7\%,\ p_1 = 67.3\%,\ s_{0\to1} = 0.0276,\ s_{1\to0} = 0.0135$

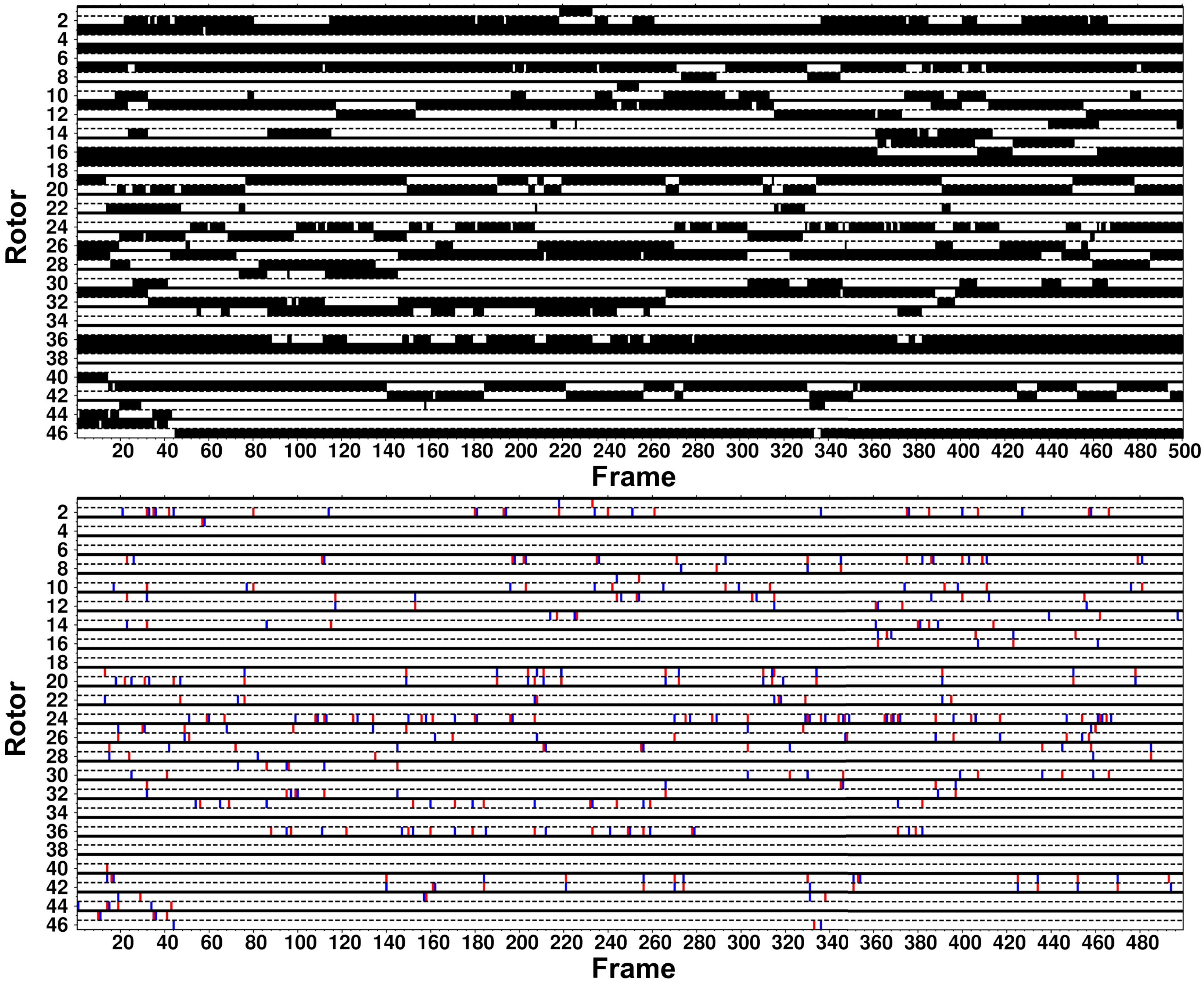

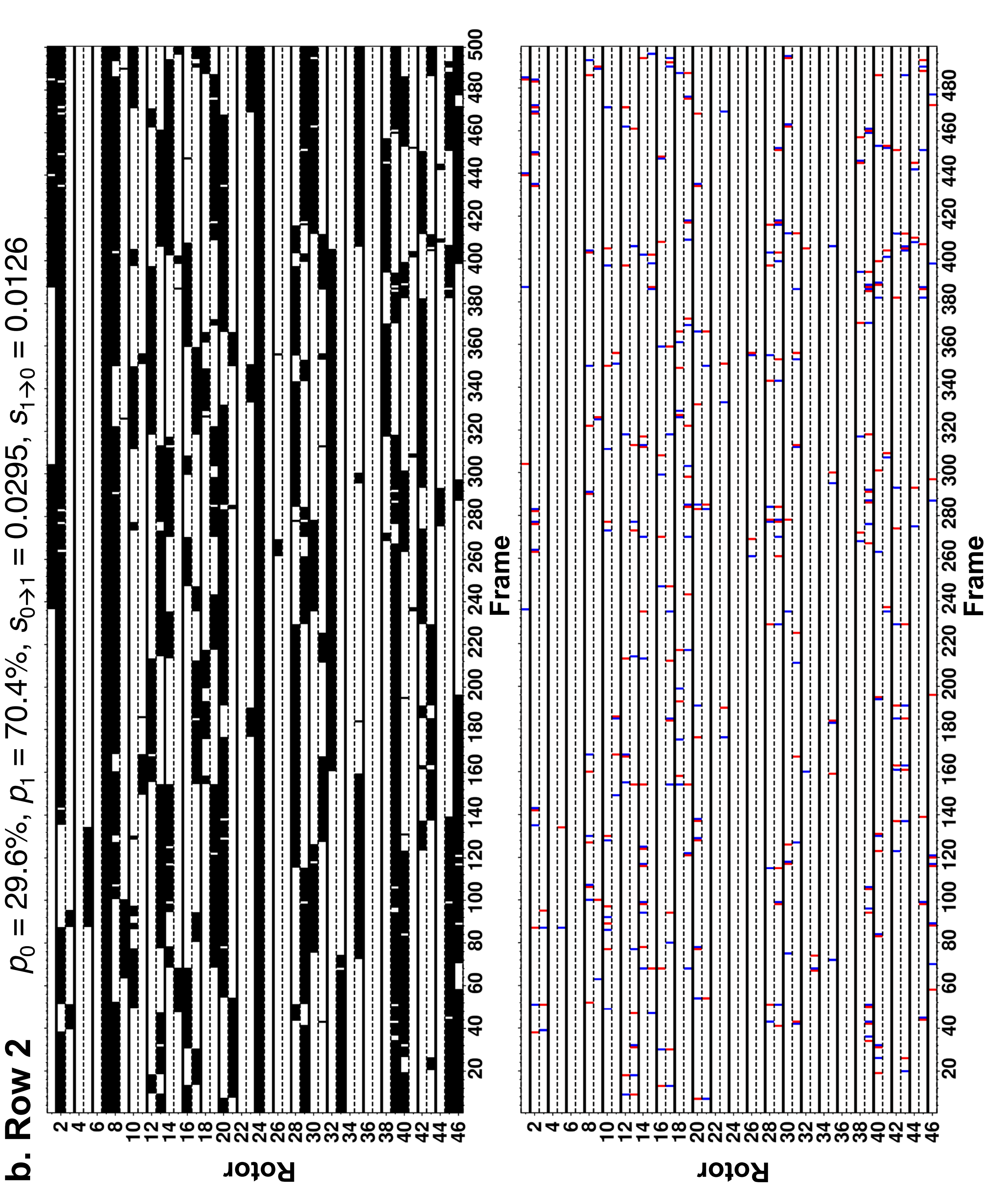
b. Row 2
$p_0 = 29.6\%$, $p_1 = 70.4\%$, $s_{0\to1} = 0.0295$, $s_{1\to0} = 0.0126$
Rotor
Frame
Rotor
Frame

c. Row 3 $p_0 = 31.4\%$, $p_1 = 68.6\%$, $s_{0\to1} = 0.0369$, $s_{1\to0} = 0.0170$

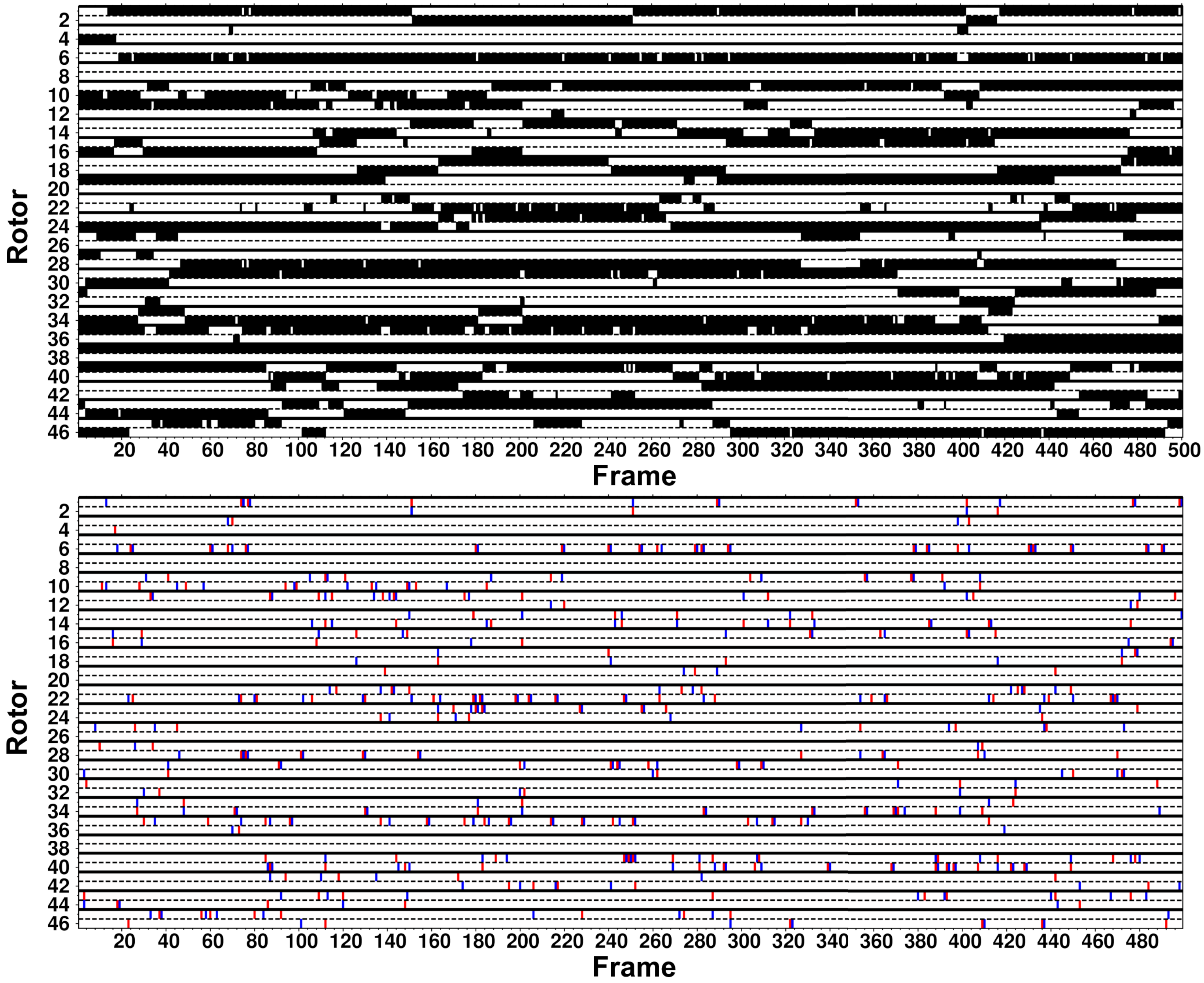

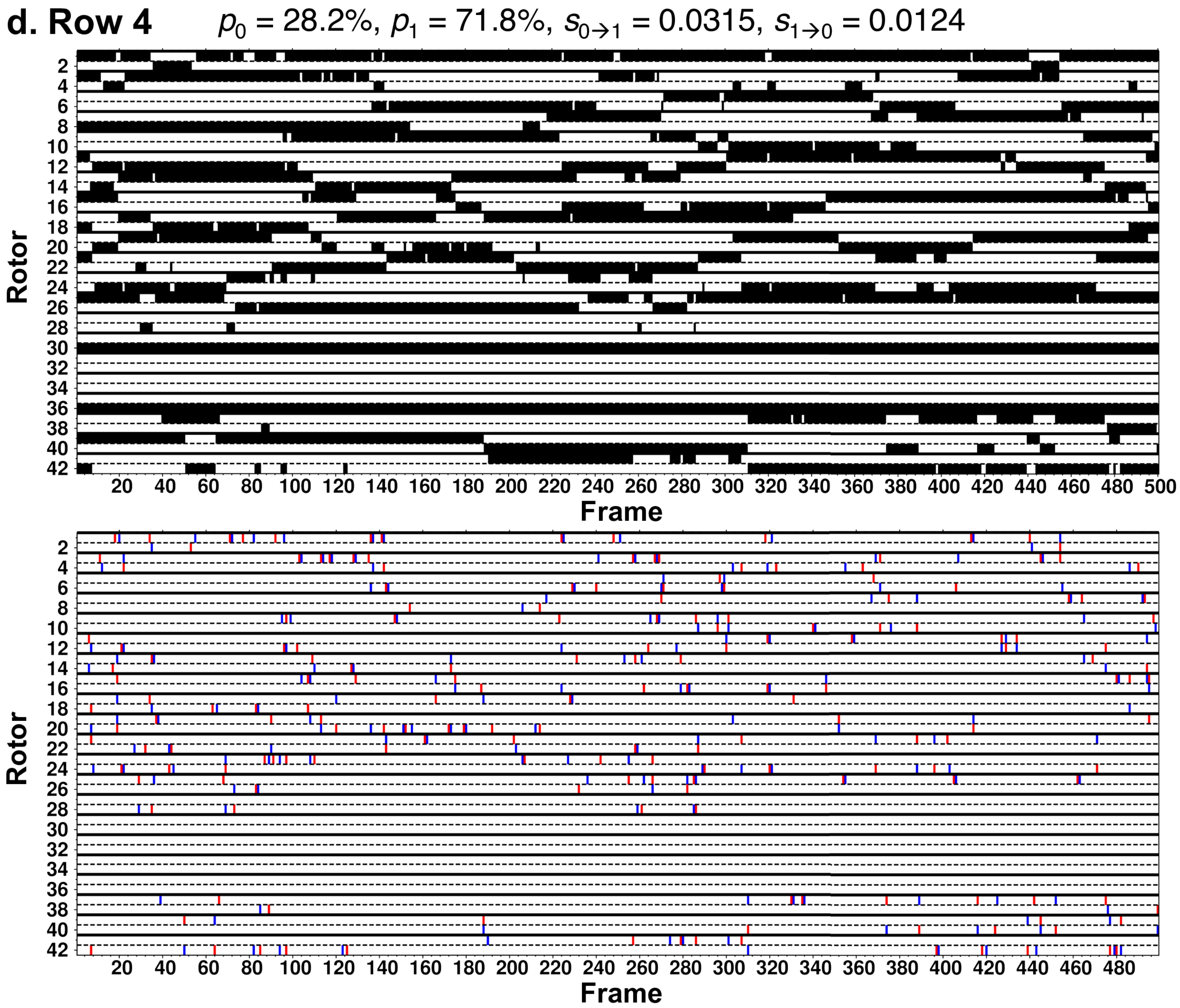

Supplementary Fig. 8. Time series and associated switching for each of the four rows of the major crystal structure formed by reacting 4-bromo-1-ethyl-2-fluorobenzene with Cu(111). For each row, the top panel shows the orientation of each rotor, where white and black indicate ethyl rotor up (1) and down (0), respectively. The bottom panel shows switching events, where red indicates switching on (0→1) and blue indicates switching off (1→0). The p_0, p_1, $s_{0\to1}$ and $s_{1\to0}$ are also specified for each row.

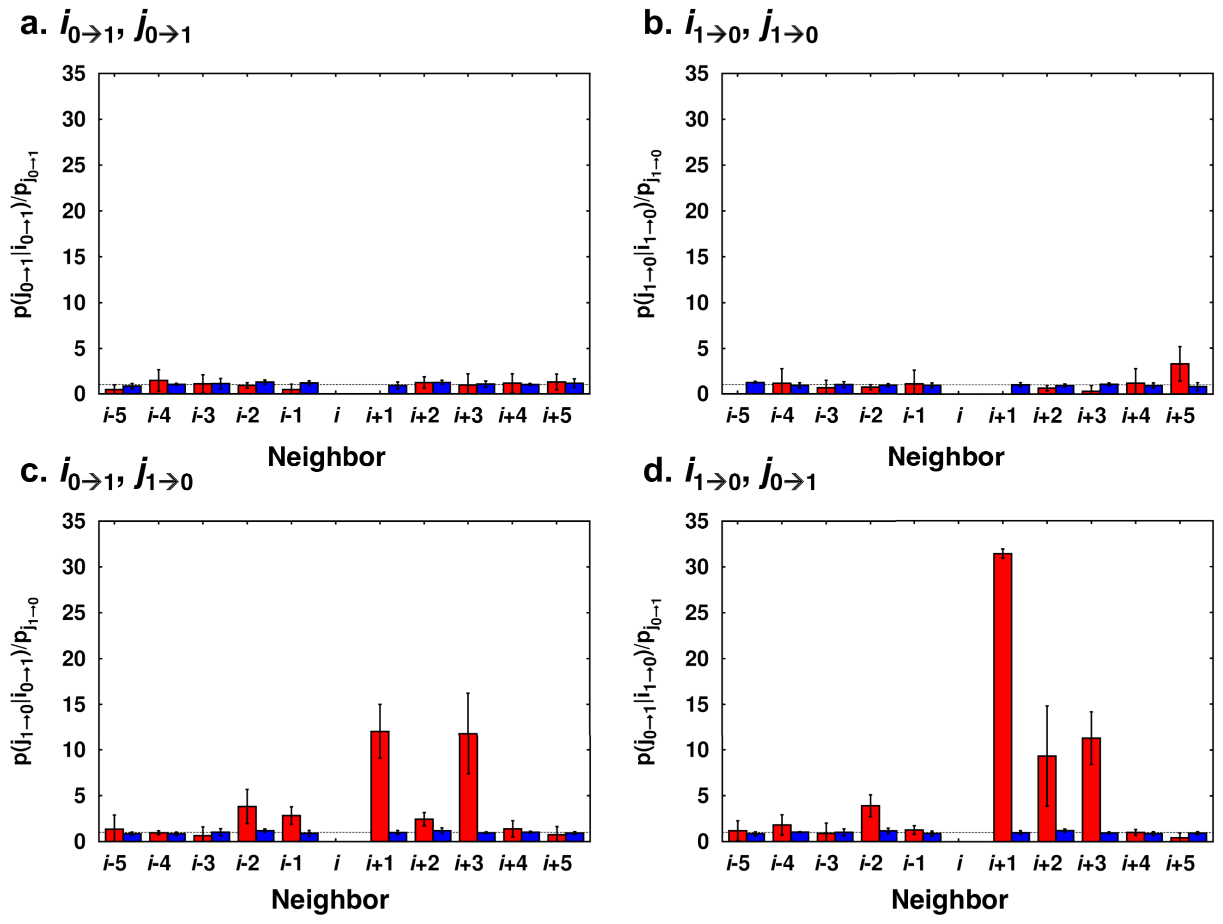

Supplementary Fig. 9. Correlation plots for each of the four categories of correlated switching in the major crystal packing structure formed by reacting 4-bromo-1-ethyl-2-fluorobenzene with Cu(111). For each category, the ratio of the conditional probability of an ethyl rotor at position *j* (*j*=*i*−5, …, *i*−1, *i*+1, …, *i*+5) switching on/off given that *i* switches to the probability of an ethyl rotor switching the on/off is calculated. The conditional probabilities for *i* and *j* both switched from 0→1 (**a**), *i* and *j* both switched from 1→0 (**b**), *i* switched from 0→1 but *j* switched from 1→0 (**c**), and *i* switched from 1→0 but *j* switched from 0→1 (**d**) are calculated. The conditional probabilities of the four categories are normalized by the $s_{0\to1}$, $s_{1\to0}$, $s_{1\to0}$ and $s_{0\to1}$, respectively. The relative position of *j* to *i* is defined so that *i*+1 is the near-neighbor of *i*, while *i*−1 is the far-neighbor of *i*. Red and blue are results from experiment and the random dataset, respectively.

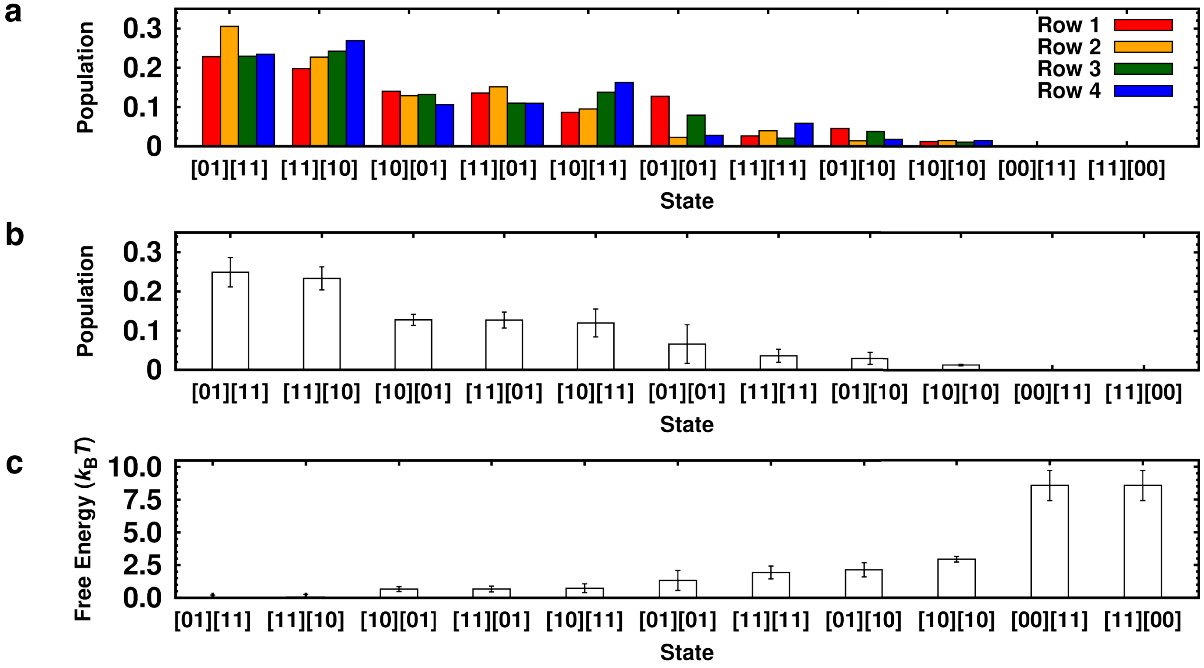

Supplementary Fig. 10. a, Populations of the two-pair microstates in the major crystal formed by reacting 4-bromo-1-ethyl-2-fluorobenzene with Cu(111). **b**, Average populations over the four rows. **c,** Free energy differences for each microstate relative to the highest populated state ([01][11]). States with populations of 0.0 are not shown.

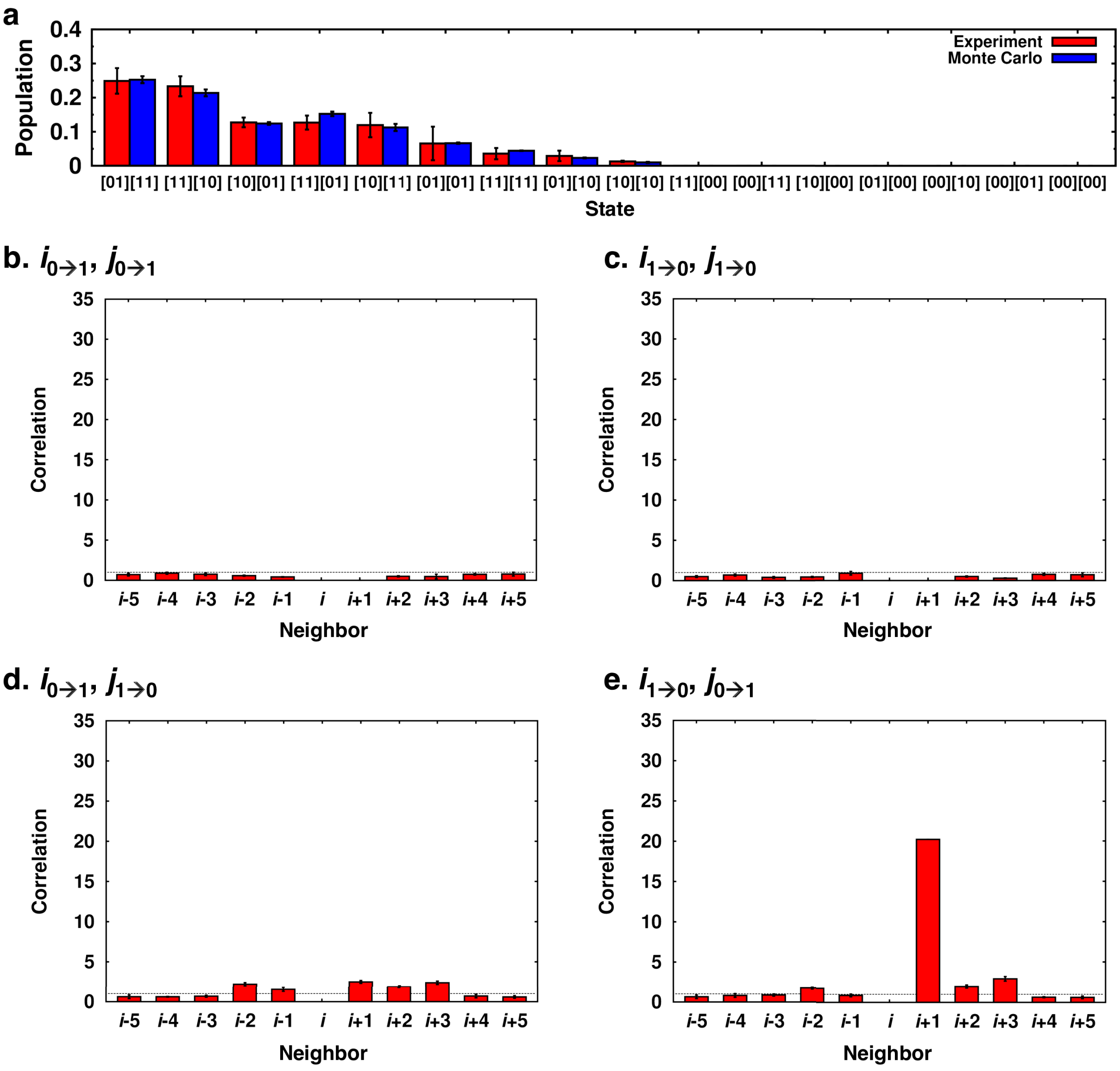

Supplementary Fig. 11. a, Populations of the 16 microstates in the MC simulation (blue) and in the experiment of the major crystal formed by reacting 4-bromo-1-ethyl-2-fluorobenzene with Cu(111) (red). **b**–**e**, Correlation plots for each of the four categories of correlated switching in MC simulation. For each category, the ratio of the conditional probability of an ethyl rotor at position *j* (*j*=*i*−5, …, *i*−1, *i*+1, …, *i*+5) switching on/off given that *i* switches to the probability of an ethyl rotor switching the on/off is calculated. The conditional probabilities for *i* and *j* both switched from 0→1 (**b**), *i* and *j* both switched from 1→0 (**c**), *i* switched from 0→1 but *j* switched from 1→0 (**d**), and *i* switched from 1→0 but *j* switched from 0→1 (**e**) are calculated and compared to $s_{0\to1}$, $s_{1\to0}$, $s_{1\to0}$ and $s_{0\to1}$, to calculate the correlation, respectively. The relative position of *j* to *i* is defined so that *i*+1 is the near-neighbor of *i*, while *i*−1 is the far-neighbor of *i*.

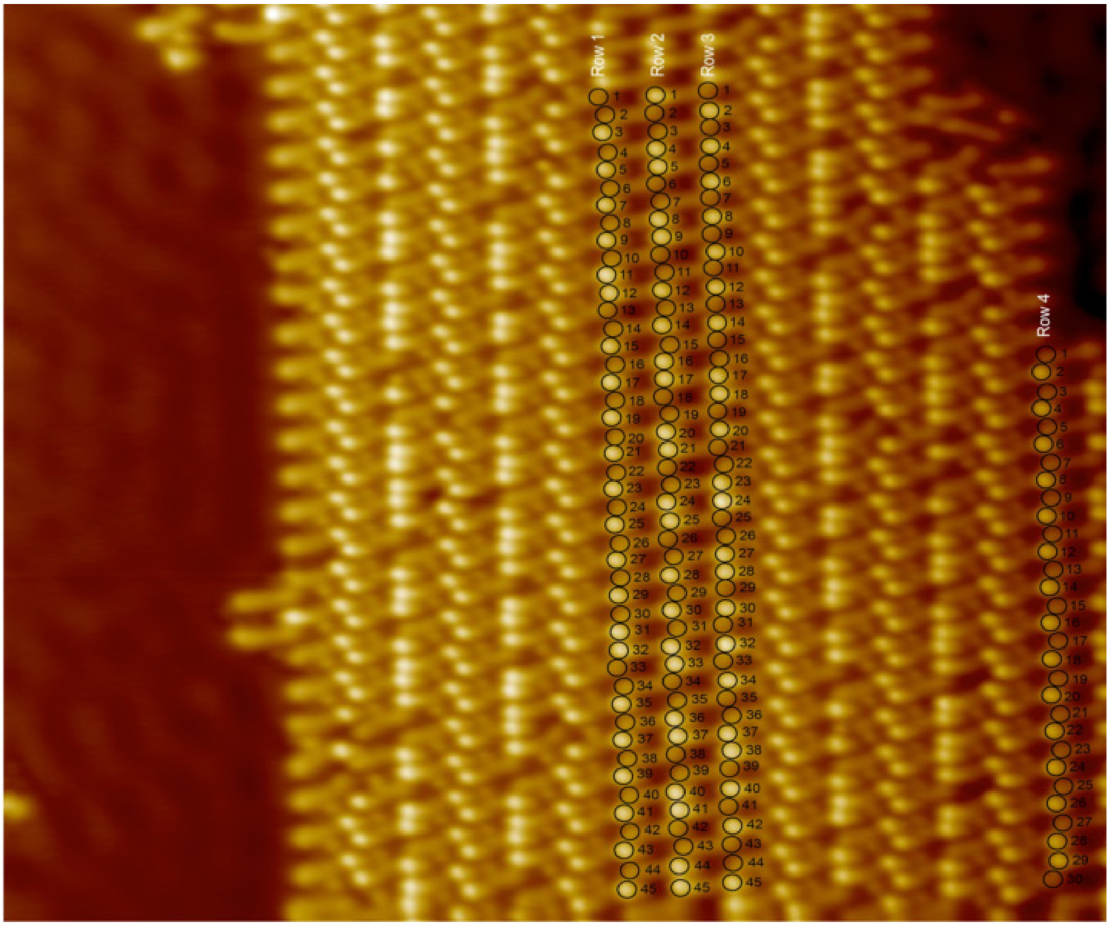

Supplementary Fig. 12. STM image of the minority crystal packing formed by reacting 4-bromo-1-ethyl-2-fluorobenzene with Cu(111) in which the ethyl rotors appear either as bright protrusions or depressions depending on their orientation. The ethyl rotors are numbered for each of the four rows analyzed. The image size and scanning conditions are 28 nm × 28 nm and −10 mV and 10 pA, respectively.

a. Row 1 $p_0 = 51.1\%$, $p_1 = 48.9\%$, $s_{0\to1} = 0.0011$, $s_{1\to0} = 0.0011$

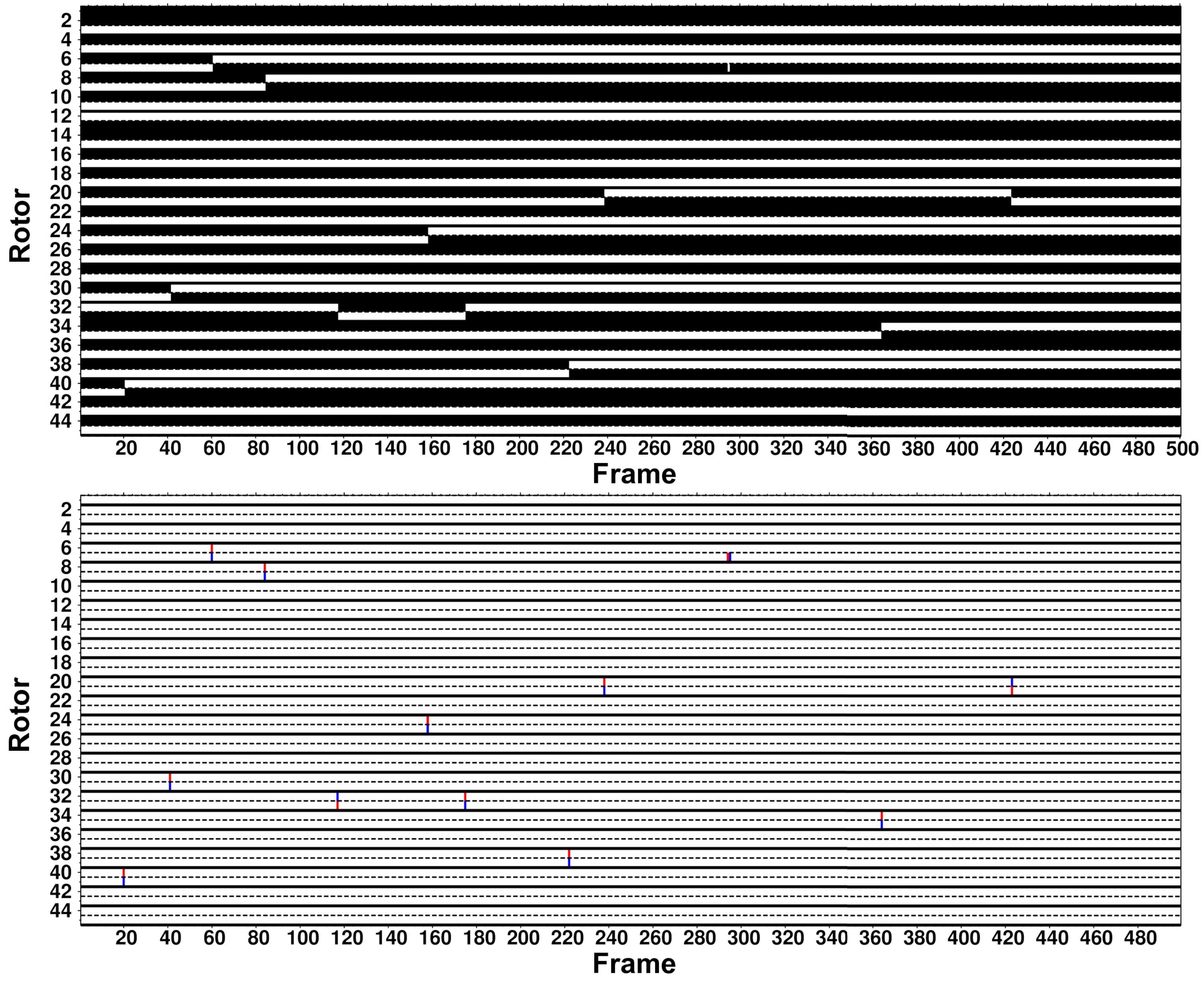

b. Row 2 $p_0 = 48.9\%$, $p_1 = 51.1\%$, $s_{0\to1} = 0.0000$, $s_{1\to0} = 0.0000$

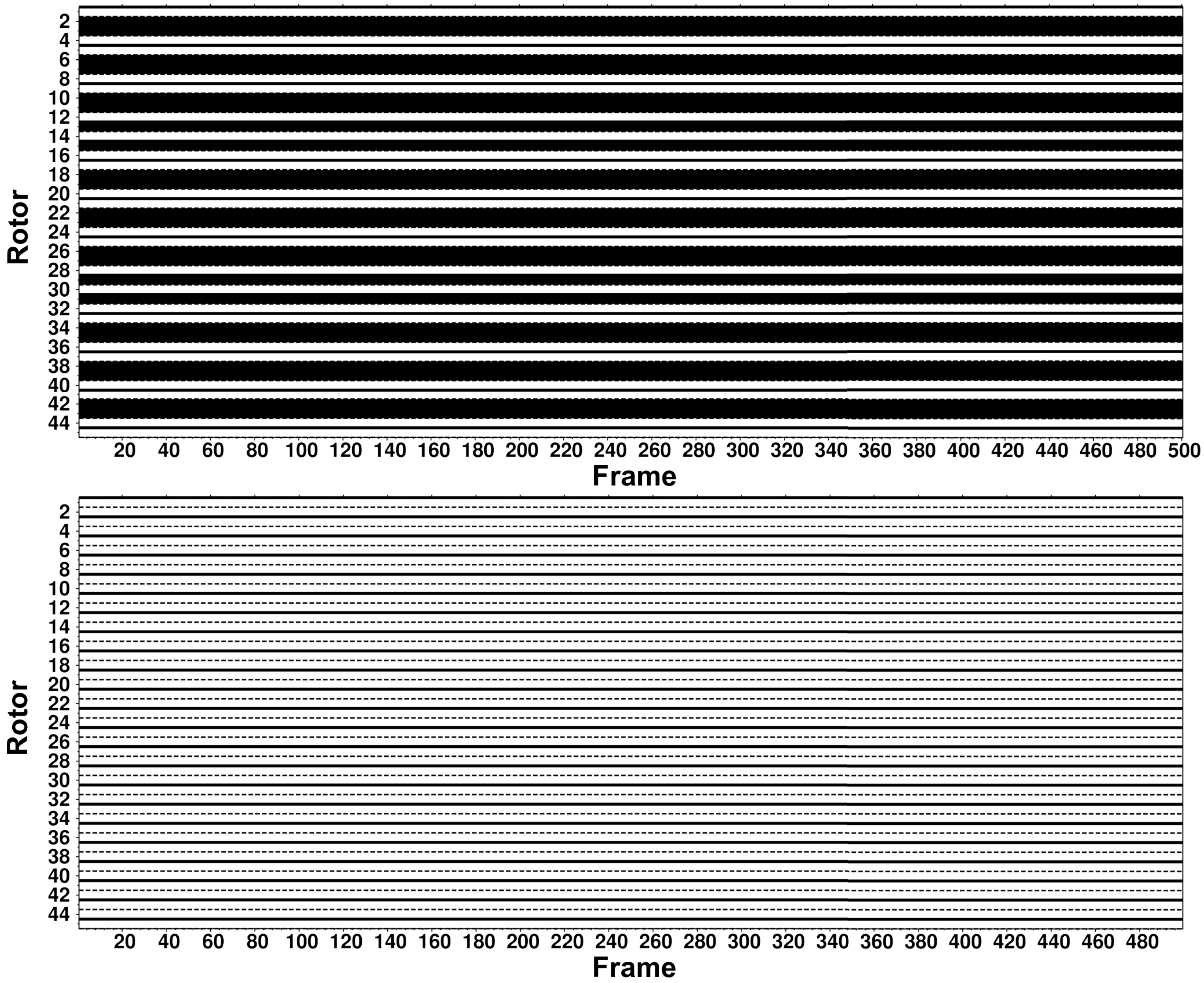

c. Row 3 $p_0 = 49.4\%$, $p_1 = 50.6\%$, $s_{0\rightarrow 1} = 0.0016$, $s_{1\rightarrow 0} = 0.0015$

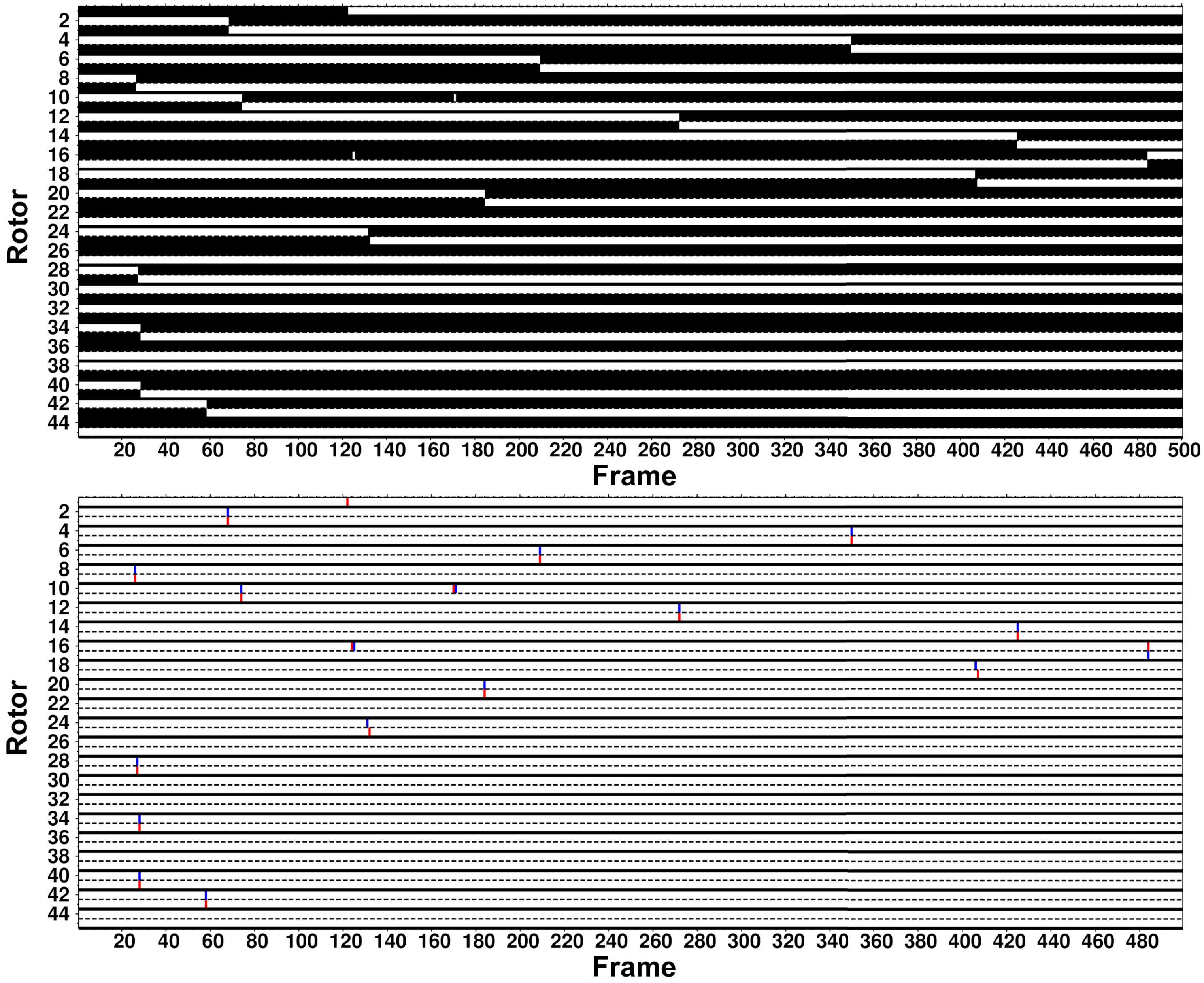

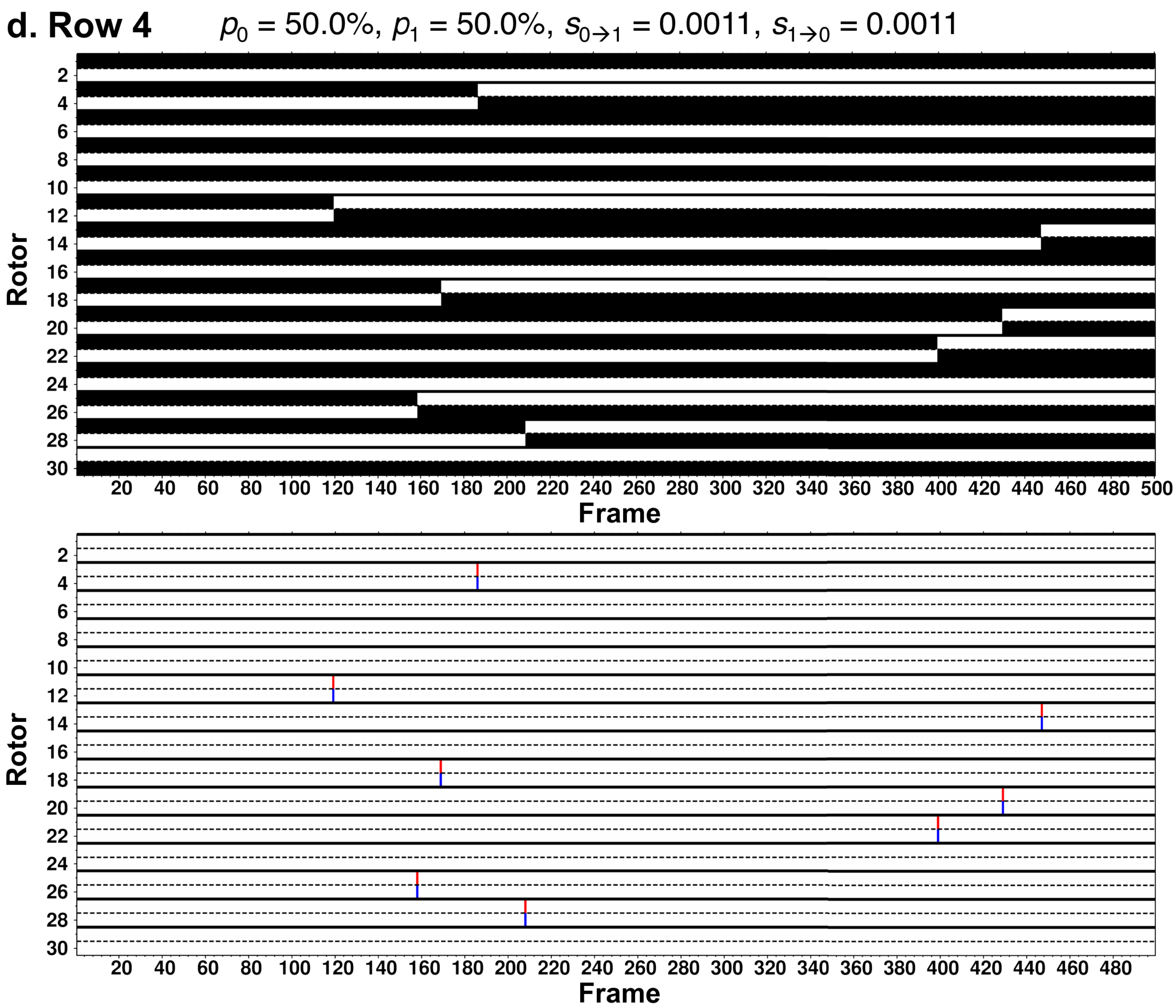

Supplementary Fig. 13. Time sequences and switching for each of the four rows in the minority crystal structure formed by reacting 4-bromo-1-ethyl-2-fluorobenzene with Cu(111). For each row, the top panel shows the ethyl orientation of each rotor, where white and black indicate ethyl rotor up (1) and down (0), respectively. The bottom panel shows switching events, where red indicates switching on (0→1) and blue indicates switching off (1→0). The p_0, p_1, $s_{0\to1}$ and $s_{1\to0}$ are also specified for each row.

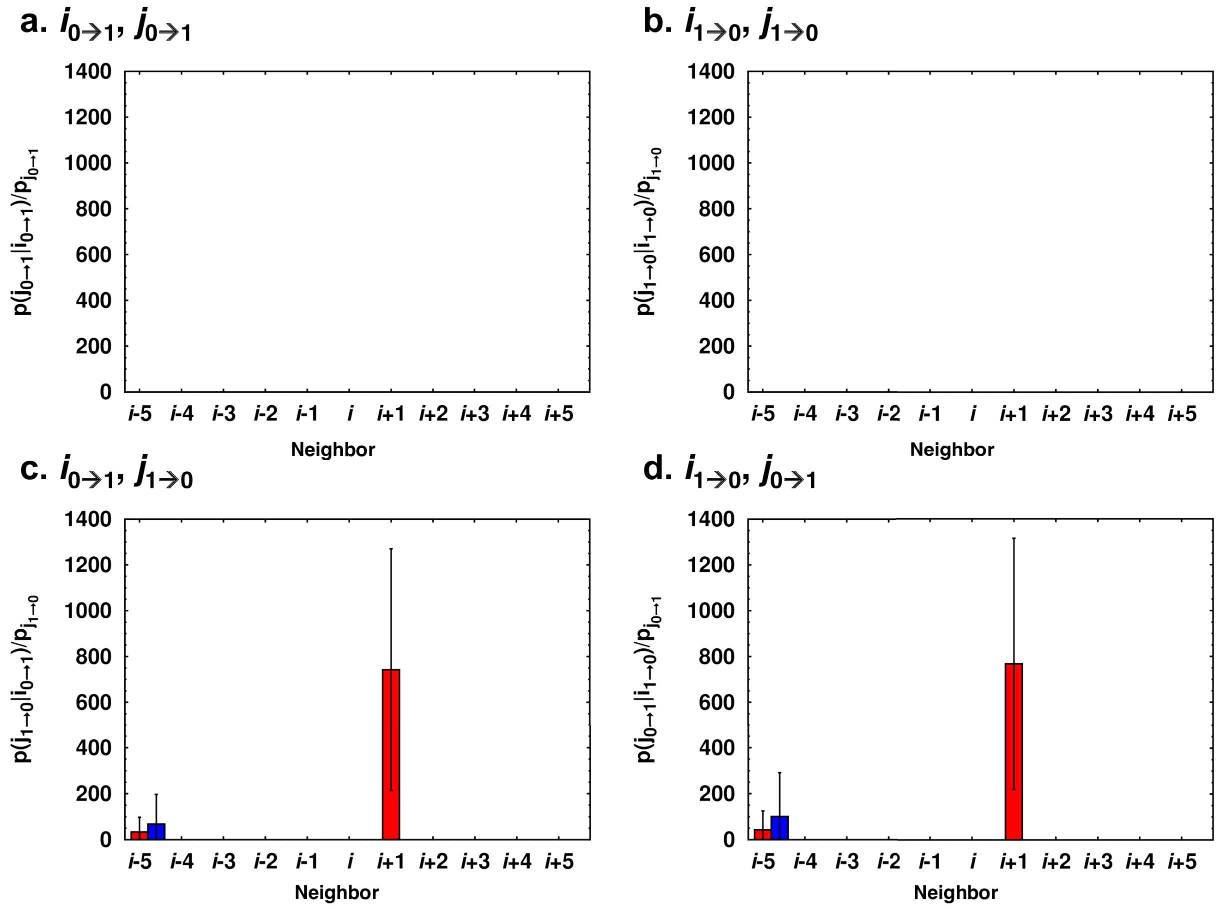

Supplementary Fig. 14. Correlation plots for each of the four categories of correlated switching in the minority crystal packing formed by reacting 4-bromo-1-ethyl-2-fluorobenzene with Cu(111). For each category, the ratio of the conditional probability of an ethyl rotor at position *j* (*j*=*i*−5, …, *i*−1, *i*+1, …, *i*+5) switching on/off given that *i* switches to the probability of an ethyl rotor switching on/off is calculated. The conditional probabilities for *i* and *j* both switched from 0→1 (**a**), *i* and *j* both switched from 1→0 (**b**), *i* switched from 0→1 but *j* switched from 1→0 (**c**), and *i* switched from 1→0 but *j* switched from 0→1 (**d**) are calculated. The conditional probabilities of the four categories are normalized by the $s_{0\to1}$, $s_{1\to0}$, $s_{1\to0}$ and $s_{0\to1}$, respectively. The relative position of *j* to *i* is defined so that *i*+1 is the near-neighbor of *i*, while *i*−1 is the far-neighbor of *i*. Red and blue are results from experiment and the random dataset.

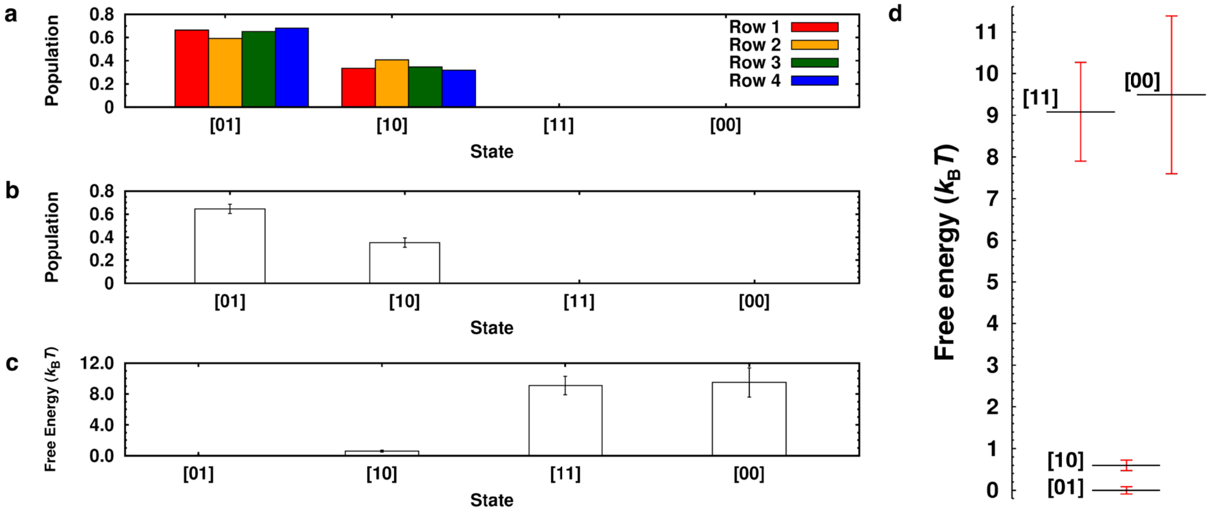

Supplementary Fig. 15. a, Populations of the one-pair microstates in the minor crystal formed by reacting 4-bromo-1-ethyl-2-fluorobenzene with Cu(111). **b,** Average populations over the four rows. **c–d,** Free energy differences for each microstate relative to the highest populated state ([01]).

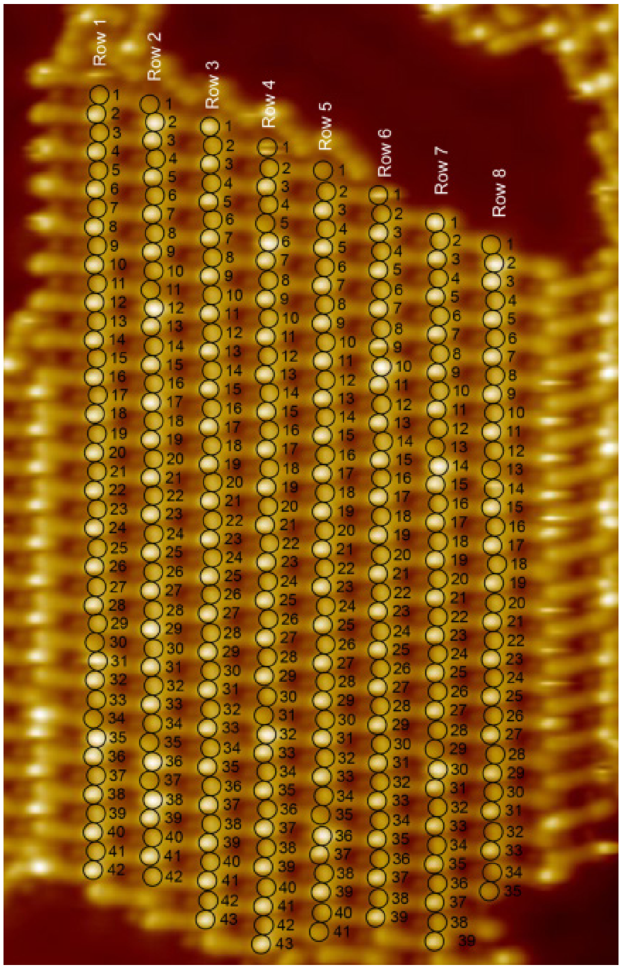

Supplementary Fig. 16. STM image of the non-fluorinated crystal formed by reacting 1-bromo-4-ethylbenzene with Cu(111). The ethyl rotors appear either as bright protrusions or depressions depending on their orientation. The ethyl rotors are numbered for each of the eight rows analyzed. The image size and scanning conditions are 16 nm × 25 nm and −30 mV and 30 pA, respectively.

a. Row 1 $p_0 = 50.0\%$, $p_1 = 50.0\%$, $s_{0\rightarrow1} = 0.0024$, $s_{1\rightarrow0} = 0.0024$

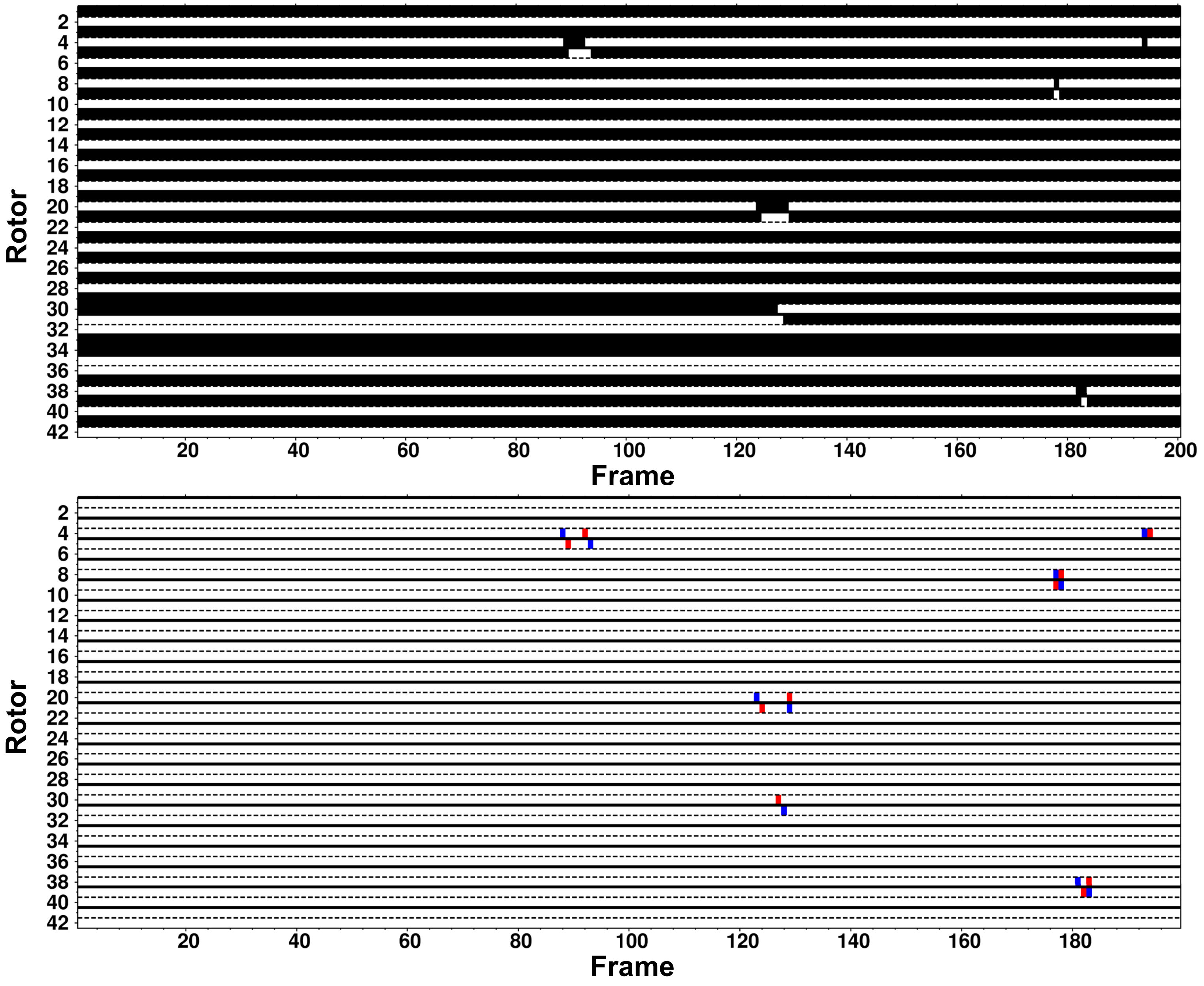

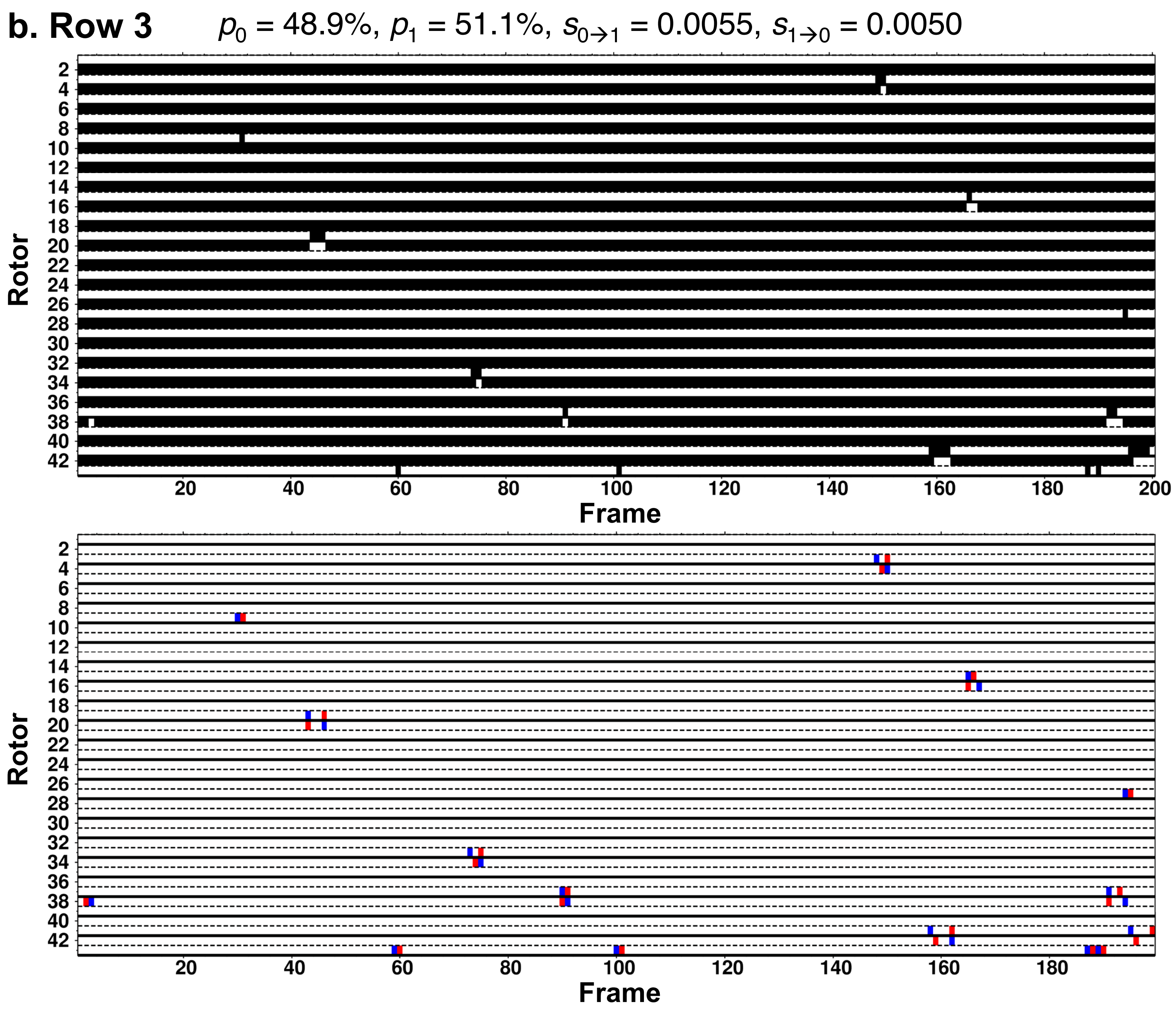
b. Row 3
p_0 = 48.9%, p_1 = 51.1%, $s_{0\to1}$ = 0.0055, $s_{1\to0}$ = 0.0050
Rotor
Frame
Rotor
Frame

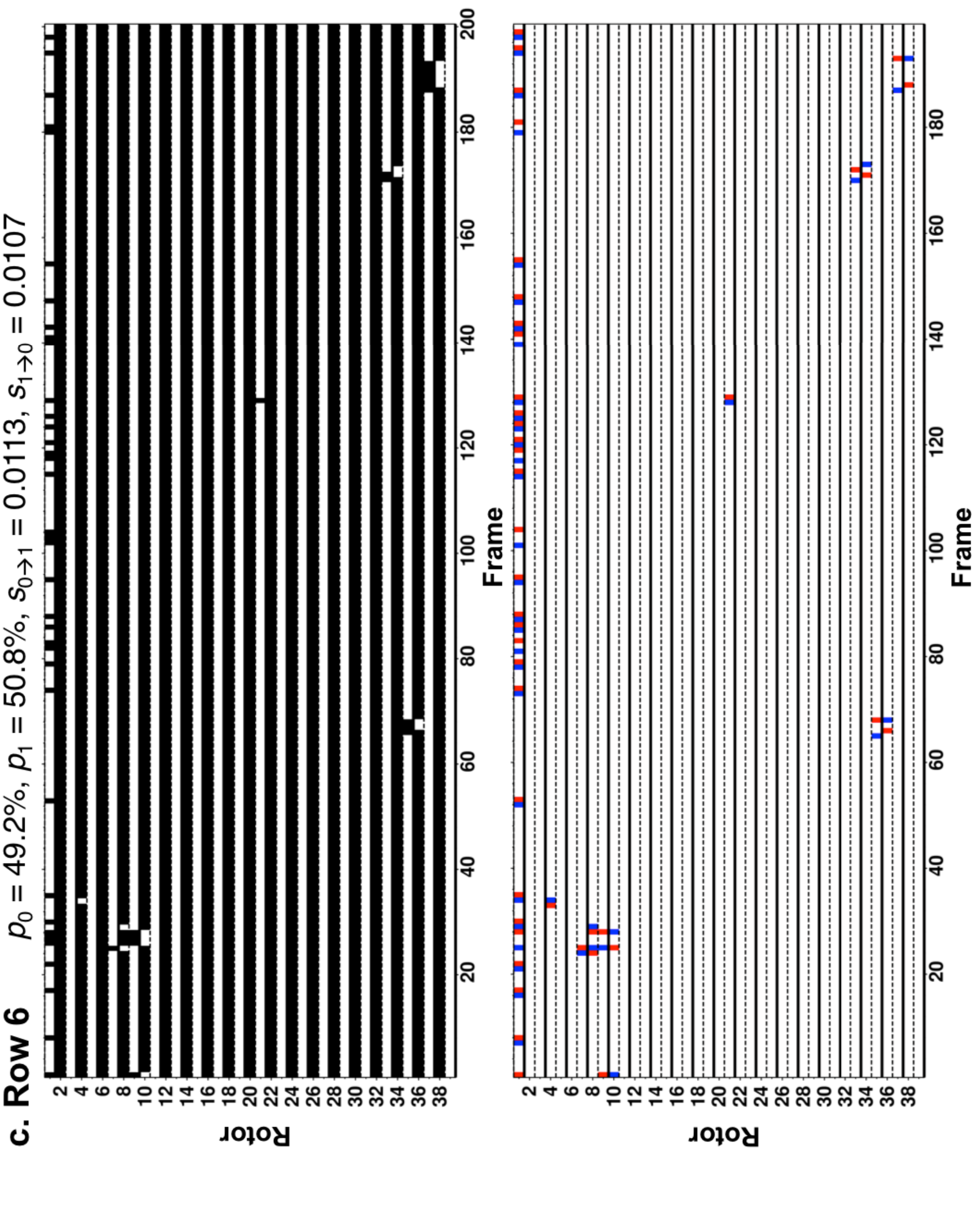
c. Row 6
$p_0 = 49.2\%$, $p_1 = 50.8\%$, $s_{0\to1} = 0.0113$, $s_{1\to0} = 0.0107$
Rotor
Frame
Rotor
Frame

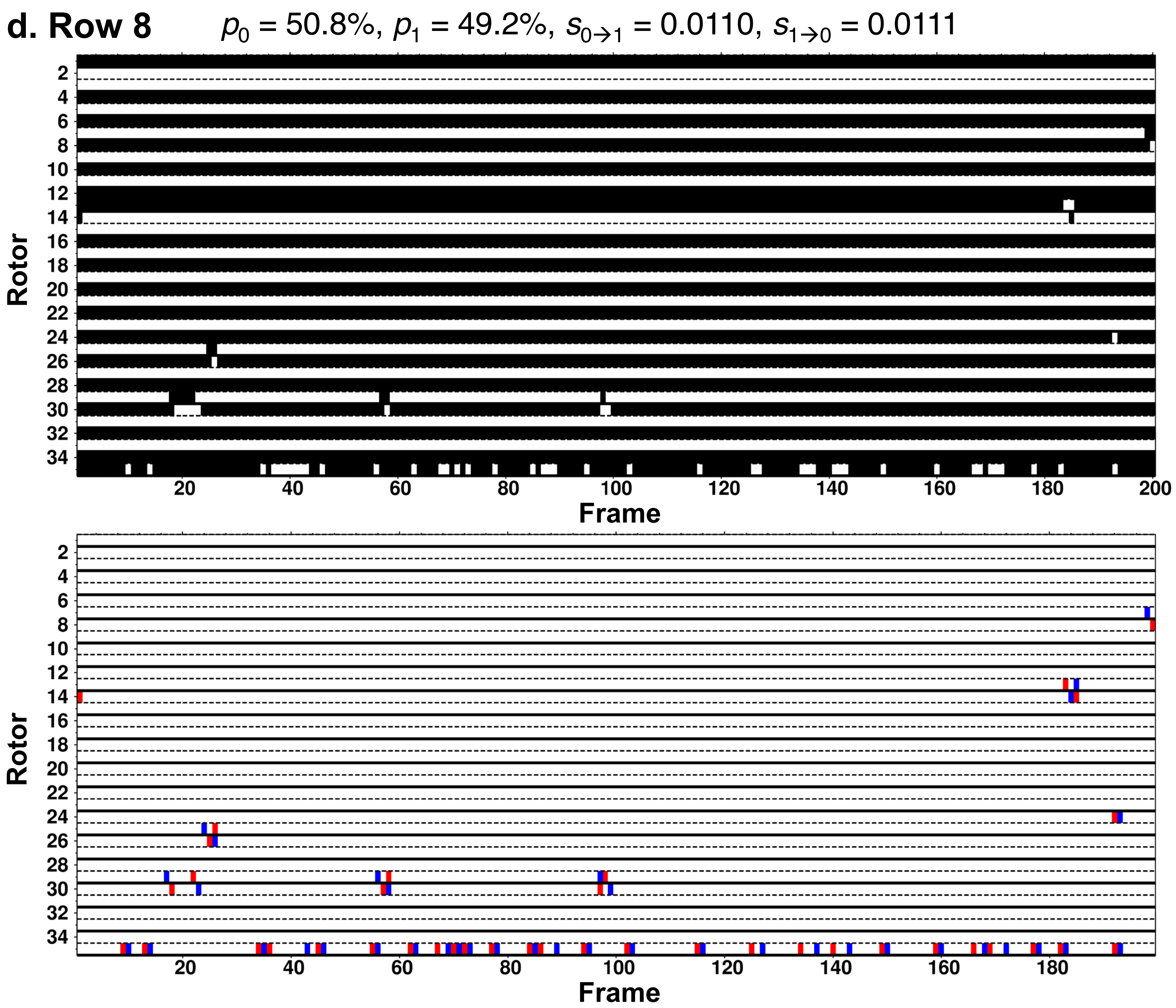

Supplementary Fig. 17. Time sequences and associated switching of four representative rows in the crystal formed by reacting 1-bromo-4-ethylbenzene with Cu(111). For each row, the top panel shows the ethyl orientation of each rotor, where white and black indicate ethyl rotor up (1) and down (0), respectively. The bottom panel shows switching events, where red indicates switching on (0→1) and blue indicates switching off (1→0). The p_0, p_1, $s_{0\to1}$ and $s_{1\to0}$ are also specified for each row.

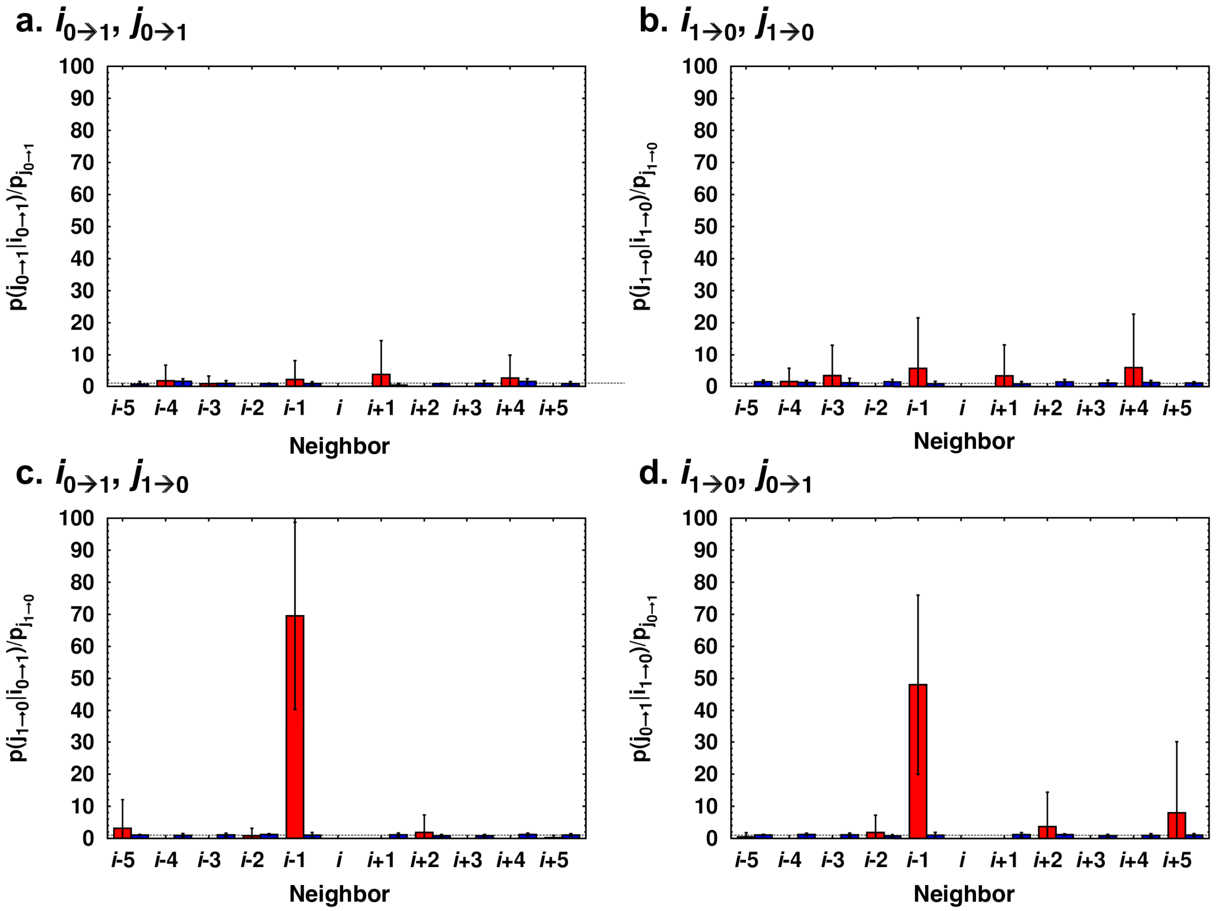

Supplementary Fig. 18. Correlation plots for each of the four cases of correlated switching in the crystal formed by reacting 1-bromo-4-ethylbenzene with Cu(111). For each category, the ratio of the conditional probability of an ethyl rotor at position j ($j=i-5$, …, $i-1$, $i+1$, …, $i+5$) switching on/off given that i switches to the probability of an ethyl rotor switching on/off is calculated. The conditional probabilities for i and j both switched from 0→1 (**a**), i and j both switched from 1→0 (**b**), i switched from 0→1 but j switched from 1→0 (**c**), and i switched from 1→0 but j switched from 0→1 (**d**) are calculated. The conditional probabilities of the four categories are normalized by the $s_{0\to1}$, $s_{1\to0}$, $s_{1\to0}$ and $s_{0\to1}$, respectively. The relative position of j to i is defined so that $i+1$ is the near-neighbor of i, while $i-1$ is the far-neighbor of i. Red and blue are results from experiment and the random dataset.

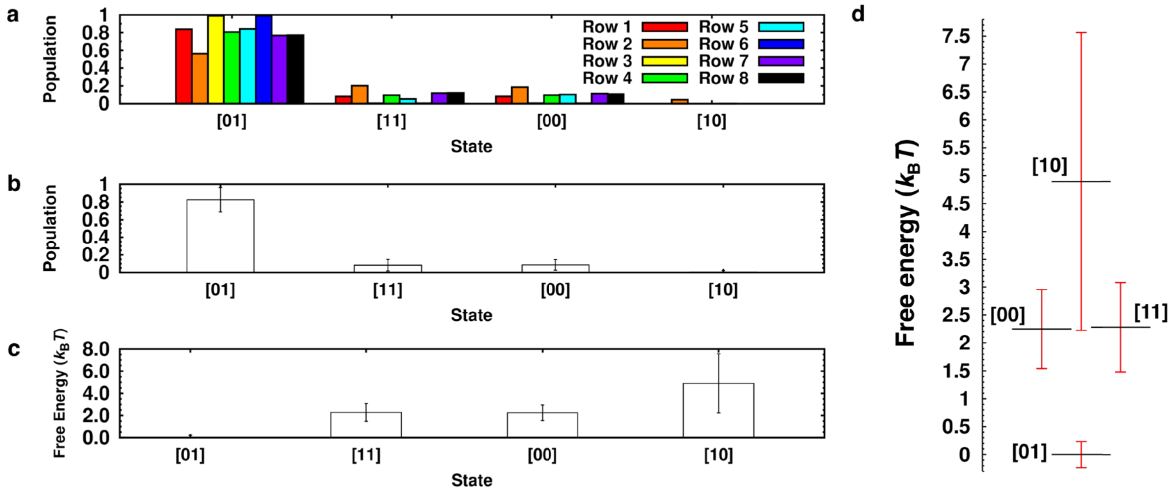

Supplementary Fig. 19. a, Population of the four microstates within one near-neighbor pair in the crystal formed by reacting 1-bromo-4-ethylbenzene with Cu(111). **b,** Average populations over the eight rows. **c–d,** Free energy differences for each microstate relative to the highest populated state ([01]).

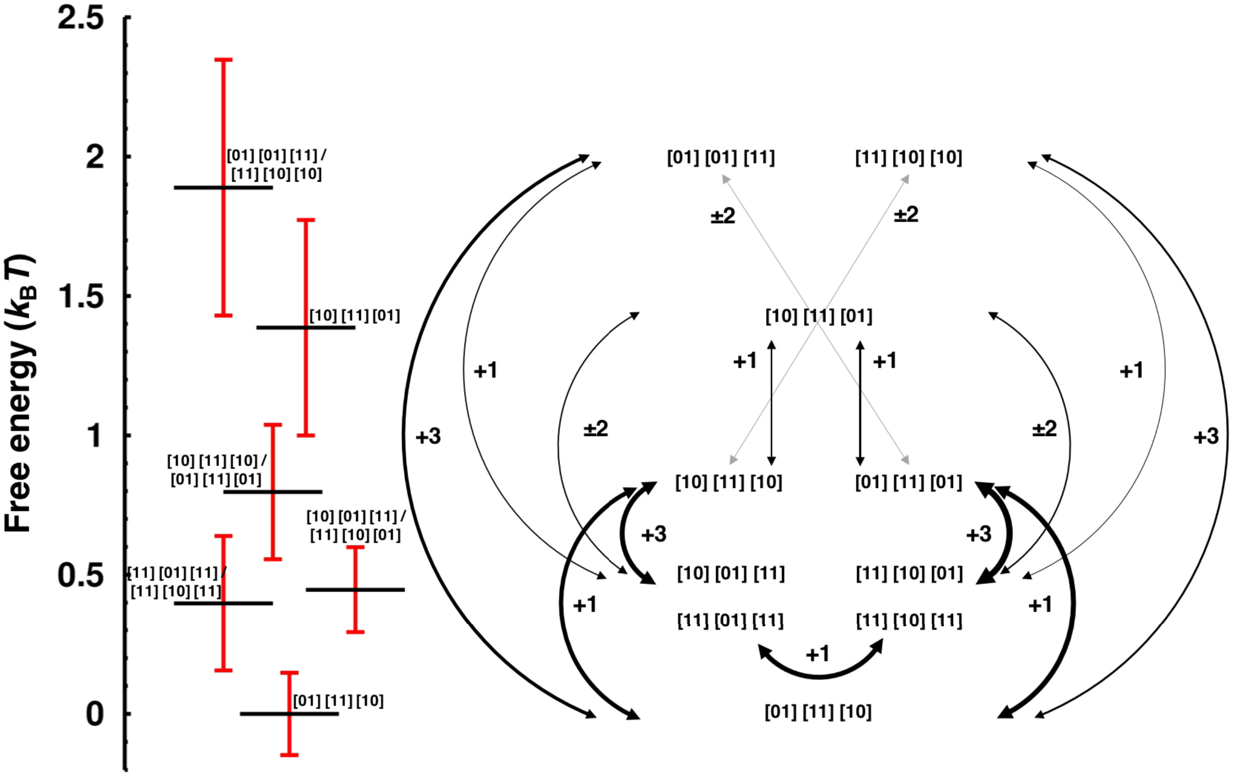

Supplementary Fig. 20. Similar to the two-pair microstate analysis previously described, we have also analyzed the data as sets of three near-neighbor pairs [*ab*][*cd*][*ef*] (a total of six ethyl rotors). **Left**: Free energies of the ten most populated [*ab*][*cd*][*ef*] microstates in the major crystal formed by reacting 4-bromo-1-ethyl-2-fluorobenzene with Cu(111). **Right**: Transitions between microstates that lead to correlated switches indicated by arrows. The thickness of the arrows is proportional to the switching flux. Labels +1, ±2 and +3 indicate (i, i+1), (i, i±2) and (i, i+3) switches that occur between microstates.

Supplementary Note 1: Control experiments to rule out thermal effects in switching

These experiments involved imaging rotor arrays for a set time, measuring the average switching probability between consecutive frames, and then moving the tip away from the array for a period of time before moving back to the original area. The images in **Supplementary Fig. 5** show 2D arrays before the tip has been moved 100 nm away for 10 minutes and then after the tip returned to the same position. Most rotors in the active rows remain in the same up/down configurations with only 6% that have switched; imaging conditions of −10 mV and 30 pA. This is in contrast to an average number of switches of >20% observed when the tip is allowed to scan the same area without moving away. Therefore, these control experiments reveal that in the absence of tunneling electrons, the array is mostly unperturbed. This is not unexpected as at 5 K the amount of thermal energy available to the rotors is 0.43 meV as compared to the calculated 50 meV torsional barrier (shown in **Supplementary Fig. 6d**).

Supplementary Note 2: Switching rate as a function of bias voltage and current

The data shown in **Supplementary Figs. 6 a–b** exhibit a strong dependence on voltage that is symmetrical about zero, indicating that the rate is dependent on the applied gap bias. Furthermore, we observe weak switching rate dependence on tunneling current. This is understood in terms of coupling of the molecular rotor's electric dipole moment with the electric field of the STM tip[2–6]. These studies have shown that the E-field can significantly reduce the activation barrier for molecular switches. Our calculations indicate that the electric dipole moment of the ethyl rotor is 0.67 Debye, which at a typical tip height of 1 nm and bias voltage of 0.2 V leads to an interaction energy ~3 meV and low electron orders N shown in panel **c** of **Supplemental Fig. 6**. This also relates to the increase in the rotor rotation rate at 40 meV in panels **a** and **b** of **Supplemental Fig. 6** which show an onset below that calculated by DFT 50 meV due the E-field of the STM tip reducing the barrier to switch.

We can also rule out the effect of saturation of the excited state that can lead to low reaction orders (N) (ref 7). Given short vibrational state lifetimes of molecules on metal surfaces (1–10 ps) the low tunneling currents used in our experiments (~10 pA) lead to the molecules spending the majority of the time (>99.9% at 10 pA) in their ground state, even assuming conservatively that each tunneling electron excites a switch.

Supplementary Note 3: Switching analysis of the major crystal formed by reacting 4-bromo-1-ethyl-2-fluorobenzene with Cu(111)

Five hundred consecutive STM frames containing four active rows were used to analyze the switching behaviors of the 2D crystal shown in **Supplementary Fig. 7**. Each ethyl rotor was denoted as a 0 (dark; ethyl down) or 1 (bright, ethyl up) based on the STM image contrast.

The time series of each of the four rows and the associated switching series are shown in **Supplementary Fig. 8**. From these data both the populations of 0 and 1 (p_0 and p_1), the switching probabilities of $0\rightarrow1$ and $1\rightarrow0$ ($s_{0\rightarrow1}$ and $s_{1\rightarrow0}$), and the overall switching probability were calculated and can be found in **Supplementary Fig. 8**. For comparison, a random dataset containing the same number of rows and rotors with matching p_0, p_1 and $s_{0\rightarrow1}$ was also generated for comparison.

As seen in **Supplementary Fig. 8**, simultaneous switches were frequently observed. To further quantify such correlation, when a switch occurred, any other simultaneous switches along with the correlation patterns and their relative positions were recorded. When simultaneous switching occurred for ethyl rotors i and j from frame N to N+1, their correlation pattern was categorized as (A) i and j both switched from $0\rightarrow1$, (B) i and j both switched from $1\rightarrow0$, (C) i switched from $0\rightarrow1$ but j switched from $1\rightarrow0$, and (D) i switched from $1\rightarrow0$ but j switched from $0\rightarrow1$. The relative position of ethyl rotor j to i (or i to j) was further defined by choosing the direction toward the near-neighbor of i (or j) as the positive direction. For example, $[\underline{1}1][1\underline{0}]\rightarrow[\underline{0}1][1\underline{1}]$ was considered simultaneous switching of i and i+3, where [ab] denotes a near-neighbor pair (a and b can be 0 or 1) and the underlines highlight the rotors that switched. Examples of correlated (i, i+1) and (i, i+3) switches are also shown in **Fig. 1d**. The conditional switching probabilities of

$i + n$ given i switched were then normalized by the average $s_{0\rightarrow 1}$ (when $i + n$ switched on) or $s_{1\rightarrow 0}$ (when $i + n$ switched off) calculated using all four rows to compute the correlations. Using the correlations from the four rows, weighted average and standard deviation (weighted by the number of ethyl rotors in each row) were calculated (**Supplementary Fig. 9**), and compared with those calculated from the random dataset (blue bars in **Supplementary Fig. 9**).

To determine the ground state, each row was first broken down into sets of two near-neighbor pairs [*ab*][*cd*] (a total of four ethyl rotors), analyzed with a sliding window. For example row 1 contained 46 ethyl rotors with near-neighbor pairs formed by rotors 1 and 2, rotors 3 and 4, etc. and thus had 23 near-neighbor pairs, and 22 sets of two near-neighbor pairs with a sliding window. For sets of two near-neighbor pairs [*ab*][*cd*], there were a total of 2^4=16 microstates. The population of each of the 16 microstates was calculated for each row (**Supplementary Fig. 10a**). To calculate the total average population for each of the 16 states, the populations obtained from the four rows were averaged and weighted by the number of two-pair sets each row had (w_i) (**Supplementary Fig. 10b**). The errors for the states were calculated by

$$\sigma_n = \sqrt{\frac{N}{N-1}\frac{\sum_{i=1}^{4} w_i (p_n^i - \bar{p}_n)^2}{\sum_{i=1}^{4} w_i}} \tag{1}$$

where p_n^i is the population of state n for a given row i, $\bar{p}_n$ is the average population of state n, and N=4.

The relative free energy of state n is then calculated based on

$$\frac{\Delta G_n}{k_B T} = -\ln \frac{\bar{p}_n}{\bar{p}_{\text{ref}}} \tag{2}$$

where $\bar{p}_{\text{ref}}$ was the population of the highest populated state (in this case state [01][11]) (**Supplementary Fig. 10c**). The errors for the states were calculated by

$$\sigma_n = \sqrt{\left(\frac{\sigma_{\bar{p}_n}}{\bar{p}_n}\right)^2 + \left(\frac{\sigma_{\bar{p}_{\text{ref}}}}{\bar{p}_{\text{ref}}}\right)^2} \tag{3}$$

.

Supplementary Note 4: Monte Carlo simulations

Monte Carlo (MC) simulations were performed and compared to the measured data. Initial on/off configurations for the four rows (each row contains 44 ethyl rotors) were randomly generated using the experimental on/off populations, p_0 and p_1; however, the [00] configuration was always rejected since it has a probability of 0.00005 in this 2D crystal. MC simulations were performed by moving from rotor 1 to 44 and first determining whether both rotors within the near-neighbor pair, as well as rotors within the previous and subsequent near-neighbor pairs would switch or not using a switching probability of 0.02. For example, for rotor *e* in [*ab*][*cd*][*ef*][*gh*][*ij*], the switching probability of rotors *c*, *d*, *f*, *g* and *h* will also be calculated. To determine whether a set of switches should be accepted or not, the change of energy before and after the switches was calculated using the microstate energies obtained from experiment (**Fig. 2f**). The energy difference is evaluated for sets of two near-neighbor pairs containing a switching rotor. For example, the energy difference between [*ab*][*cd*][*ef*][*gh*][*ij*] and [*ab*][*c'd'*][*e'f'*][*g'h'*][*ij*] (*c*, *d*, *e*, *f*, *g* and *h* switched to *c'*, *d'*, *e'*, *f'*, *g'* and *h'*, respectively) was $\Delta G = G([ab][c'd']) + G([c'd'][e'f']) + G([e'f'][g'h']) + G([g'h'][ij]) - G([ab][cd]) - G([cd][ef]) - G([ef][gh]) - G([gh][ij])$. If $\Delta G < 0$, the switch was accepted. If $\Delta G \geq 0$, the Boltzmann factor

$$k = e^{-\frac{\Delta G}{k_B T}} \tag{4}$$

was computed and compared to a random number ρ ranging from 0 to 1; if $k \geq \rho$, the switch was accepted; otherwise the switch was rejected. The results of four sets MC simulations each with 5,000 steps are shown in **Supplementary Fig. 11**.

Supplementary Note 5: Switching analysis of the minority crystal formed by reacting 4-bromo-1-ethyl-2-fluorobenzene with Cu(111)

Five hundred consecutive STM frames containing four active rows were used to analyze the switching behaviors of the minority crystal formed by reacting 4-bromo-1-ethyl-2-fluorobenzene with Cu(111) shown in **Supplementary Fig. 12**. Similar to the major crystal structure, ethyl rotor states were assigned 0 or 1 based on the brightness of the image. The time series for each of the four rows and the associated switching series are shown in **Supplementary Fig. 13**. The

correlations were calculated for the four categories where *i* switches from 0→1 or 1→0 and *j* switches from 0→1 or 1→0 (**Supplementary Fig. 14**). This minor crystal structure formed by reacting 4-bromo-1-ethyl-2-fluorobenzene with Cu(111) has an average ethyl rotor up (0) to down (1) population of 50:50 ± 1%. As seen in **Supplementary Fig. 13** and **Supplementary Fig. 14**, the majority of the correlated switches were correlated (*i*, *i*+1) switches.

To further understand this crystal packing, the ethyl rotors were broken down into sets of one near-neighbor pair to determine the ground state. There are four possible ground state configurations: [00], [01], [10] and [11]. The population of each of the four states for each row is shown in **Supplementary Fig. 15a**. The average population and their relative free energies are shown in **Supplementary Figs. 15b and 15c**. The relative free energy and associated error of each state was calculated based on **Eqns. 1–3**. The relative free energies of the four microstates are also shown in **Supplementary Fig. 15d**.

Supplementary Note 6: Switching analysis of the non-fluorinated crystal, formed by reacting 1-bromo-4-ethylbenzene with Cu(111)

To analyze the switching behavior of the non-fluorinated crystal formed by reacting 1-bromo-4-ethylbenzene with Cu(111), two hundred consecutive STM frames containing eight active rows were used (**Supplementary Fig. 16**). The time series of four representative rows along with the associated switching series are shown in **Supplementary Fig. 17**. The population of up vs. down states was 50:50 ± 1% for this crystal. From the time series in **Supplementary Fig. 17**, correlated (*i*, *i*–1) switches were commonly observed. The correlations were calculated for the four categories where *i* switches from 0→1 or 1→0 and *j* switches from 0→1 or 1→0 (**Supplementary Fig. 18**).

To further understand this crystal packing, the ethyl rotors were broken down into sets of one near-neighbor pair to determine the ground state, similar to the minor crystal packing. The population of each of the four states for each row is shown in **Supplementary Fig. 19a**. The eight rows were then averaged and weighted by the number of sets of one nearest-neighbor pair

(**Supplementary Fig. 19b**). The relative free energies of the four microstates are shown in **Supplementary Figs. 19c and 19d**.

Supplementary References

1. Frisch, M. J., Trucks, G. W., Schlegel, H. B., Scuseria, G. E., Robb, M. A., Cheeseman, J. R., Scalmani, G., Barone, V., Mennucci, B., Petersson, G. A., Nakatsuji, H., Caricato, M. & Li, X. Gaussian 09, Revision E.01. (2009).

2. Mo, Y. W. Reversible rotation of antimony dimers on the silicon (001) surface with a scanning tunneling microscope. *Science* **261,** 886–888 (1993).

3. Alemani, M., Peters, M. V, Hecht, S., Rieder, K.-H., Moresco, F. & Grill, L. Electric Field-Induced Isomerization of Azobenzene by STM. *J. Am. Chem. Soc.* **128,** 14446–14447 (2006).

4. Carpinelli, J. M. & Swartzentruber, B. S. Direct measurement of field effects on surface diffusion. *Phys. Rev. B* **58,** R13423–R13425 (1998).

5. Ohto, T., Rungger, I., Yamashita, K., Nakamura, H. & Sanvito, S. Ab initio theory for current-induced molecular switching: Melamine on Cu(001). *Phys. Rev. B* **87,** 205439 (2013).

6. Fu, Q., Luo, Y., Yang, J. & Hou, J. Understanding the concept of randomness in inelastic electron tunneling excitations. *Phys. Chem. Chem. Phys.* **12,** 12012–12023 (2010).

7. Parschau, M., Rieder, K.-H., Hug, H. J. & Ernst, K.-H. Single-Molecule Chemistry and Analysis: Mode-Specific Dehydrogenation of Adsorbed Propene by Inelastic Electron Tunneling. *J. Am. Chem. Soc.* **133,** 5689–5691 (2011).
